# Optical control of the β_2_-adrenergic receptor with opto-prop-2: A *cis*-active azobenzene analog of propranolol

**DOI:** 10.1016/j.isci.2022.104882

**Published:** 2022-08-05

**Authors:** Reggie Bosma, Nicola C. Dijon, Yang Zheng, Hannes Schihada, Niels J. Hauwert, Shuang Shi, Marta Arimont, Rick Riemens, Hans Custers, Andrea van de Stolpe, Henry F. Vischer, Maikel Wijtmans, Nicholas D. Holliday, Diederik W.D. Kuster, Rob Leurs

**Affiliations:** 1 Division of Medicinal Chemistry, Faculty of Science, Amsterdam Institute for Molecular and Life Sciences, Vrije Universiteit Amsterdam, 1081 Amsterdam, the Netherlands; 2 School of Life Sciences, The Medical School, Queen’s Medical Centre, University of Nottingham, Nottingham, UK; 3 Section of Receptor Biology & Signaling, Department of Physiology & Pharmacology, Karolinska Institutet, 17165 Stockholm, Sweden; 4 Department of Pharmaceutical Chemistry, Philipps-University Marburg, Marburg, Germany; 5 Amsterdam UMC Location Vrije Universiteit Amsterdam, Physiology, De Boelelaan 1117, Amsterdam, the Netherlands; 6 Amsterdam Cardiovascular Sciences, Heart Failure & Arrhythmias, Amsterdam, the Netherlands

**Keywords:** Photomedicine, Biochemical engineering, Biochemical research method

## Abstract

In this study, we synthesized and evaluated new photoswitchable ligands for the beta-adrenergic receptors β_1_-AR and β_2_-AR, applying an azologization strategy to the first-generation beta-blocker propranolol. The resulting compounds (Opto-prop-1, -2, -3) have good photochemical properties with high levels of light-induced *trans*-*cis* isomerization (>94%) and good thermal stability (*t*_1/2_ > 10 days) of the resulting *cis*-isomer in an aqueous buffer. Upon illumination with 360-nm light to PSS_*cis*_, large differences in binding affinities were observed for photoswitchable compounds at β_1_-AR as well as β_2_-AR. Notably, Opto-prop-2 (VUF17062) showed one of the largest optical shifts in binding affinities at the β_2_-AR (587-fold, *cis*-active), as recorded so far for photoswitches of G protein-coupled receptors. We finally show the broad utility of Opto-prop-2 as a light-dependent competitive antagonist of the β_2_-AR as shown with a conformational β_2_-AR sensor, by the recruitment of downstream effector proteins and functional modulation of isolated adult rat cardiomyocytes.

## Highlights

• A photoswitchable antagonist of the β_2_-AR was developed: Opto-prop-2
• β_2_-AR binding affinity of the light-induced *cis*-Opto-prop-2 is 578-fold stronger
• Opto-prop-2 allowed dynamic control of β_2_-AR antagonism
• Opto-prop-2 allowed light-dependent modulation of cardiomyocyte function

## Graphical abstract

**Figure:**
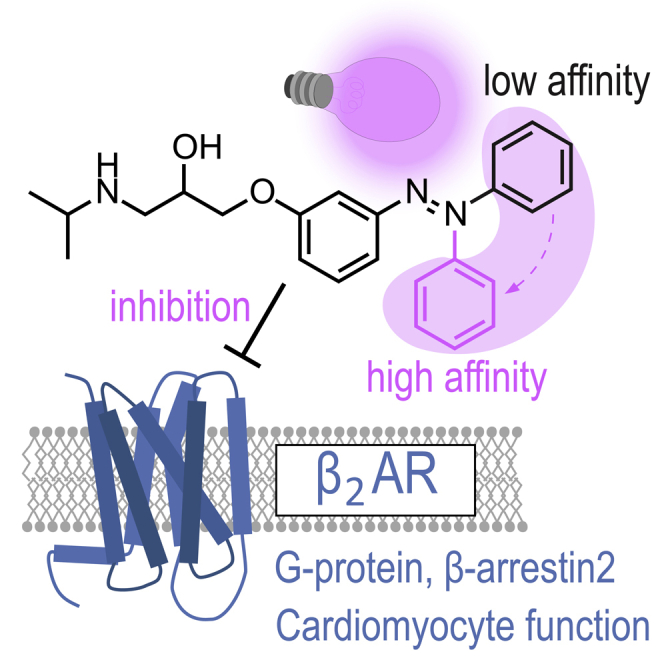

## Introduction

Modulating G protein-coupled receptor (GPCR) activity with small-molecule drugs has historically been a successful therapeutic strategy, with 33% of all small-molecule drugs targeting this class of proteins (Santos et al., 2017). Drugs are typically designed to have high and sustained occupancy of these receptors *in vivo*. However, for an in-depth exploration of GPCRs in (patho)physiology, it can be beneficial to have more dynamic control. Optogenetics has e.g. become a popular experimental approach for the flexible control of neurophysiology (Deisseroth, 2015) but also cardiophysiology (Entcheva and Kay, 2021). Optogenetics makes use of transgenic biological systems with light-sensitive proteins that drive a specific signaling pathway, allowing a high level of spatial and temporal control of signaling using light (Deisseroth, 2015; Entcheva and Kay, 2021).

**Figure 1:**
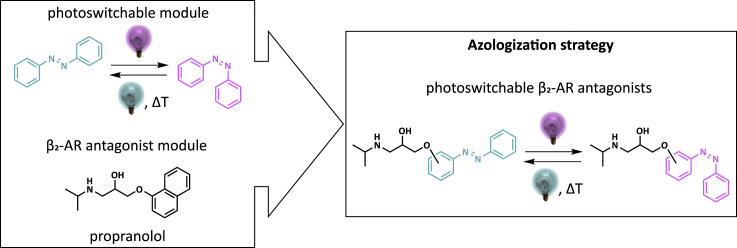
Design of propranolol-based photoswitchable inhibitors of the β_2_-AR Azobenzene was selected as a photoswitchable moiety and propranolol was taken as the standard antagonist of the β_2_-AR. Photoswitchable antagonists were designed by azologization, replacing the naphthalene ring of propranolol with an azobenzene. The propranolol side chain was positioned at either the *ortho*-, *meta*-, or *para*-position of the azobenzene.

To attenuate cellular signaling using endogenously expressed receptor proteins, recently, complementary photopharmacology strategies have emerged to design ligands with light-tunable receptor interaction (Fuchter, 2020; Hüll et al., 2018; Lerch et al., 2016; Wijtmans et al., 2022). An effective photopharmacological strategy for the optical control of GPCRs is so-called photoswitching, in which GPCR ligands are designed to incorporate a photochromic moiety, that isomerizes upon illumination. Although a number of photochromic moieties are known, for photochromic ligands targeting family A GPCRs the azobenzene-moiety constitutes around 80% of the cases (Wijtmans et al., 2022). Azobenzene (Figure 1) can reversibly switch from its thermodynamically stable *trans*-isomer to its *cis*-isomer under the influence of light (Beharry and Woolley, 2011). This isomerization of the azobenzene moiety constitutes a significant change in the shape and physicochemical properties. Consequently, the incorporation of an azobenzene group into GPCR ligands can often impose isomer-dependent differences in receptor interactions. Indeed, for various GPCRs, it has been shown that the use of azobenzene as a photochromic moiety in small-molecule ligands is a successful strategy for the photopharmacological control of GPCR activity (Berizzi and Goudet, 2020; Ricart-Ortega et al., 2019; Wijtmans et al., 2022).

The β_2_-adrenergic receptor (β_2_-AR) is a hallmark family A GPCR and has been extensively studied in the field of pharmacology, cell signaling, and structural biology (Weis and Kobilka, 2018). The ubiquitously expressed β_2_-AR recognizes the endogenous cell signaling molecules epinephrine and norepinephrine and as such, this GPCR is an important effector of the sympathetic nervous system. The β_2_-AR is well known to e.g. affect the contractility of smooth muscle cells in the lungs and the function of the heart. Agonists of the β_2_-AR are therefore used to target the lungs in the treatment of asthma (Barnes, 2011; Gorre and Vandekerckhove, 2010; Najafi et al., 2016b). Upon agonist activation, the β_2_-AR mediates intracellular signaling via heterotrimeric G_s_-proteins, which leads to increased cAMP levels and subsequent activation of protein kinase A, an important signaling cascade for, among others, smooth muscle relaxation (Barnes, 2011; Najafi et al., 2016b). The activation of the β_2_-AR also results in the recruitment of β-arrestin2, which prevents G protein coupling and facilitates receptor internalization over time (Lohse et al., 1990; Najafi et al., 2016b). As the β_2_-AR is ubiquitously expressed throughout the body (Uhlen et al., 2015), a photoswitchable β_2_-AR ligand to control β_2_-AR activation could be a valuable tool to study the spatiotemporal control of β_2_-AR function in various biological systems.

In this study, we present our work on azobenzene-derived propranolol analogs. Whereas our work was ongoing, Duran-Corbera et al. (2020) have published related β_2_-AR compounds, also based on propranolol. With our complementary strategy, we obtained a novel, thermally stable, *cis*-active photoswitchable β_2_-AR ligand named Opto-prop-2. All compounds efficiently isomerize to the *cis-*state resulting in light-tunable affinities at the β_1_-AR and the β_2_-AR. Opto-prop-2 shows one of the largest optical shifts in binding affinities for family A GPCRs (587-fold) (Wijtmans et al., 2022). We also show that our new photoswitchable compound can be used as a tool for the photopharmacological control of endogenously expressed beta-adrenergic receptors. Preliminary accounts of this work have been presented at the BPS Pharmacology Winter meeting 2019 (Bosma, 2019) and Pharmacology 2020 (Leurs, 2020).

## Results

### Synthesis and photochemical characterization photoswitchable ligands opto-prop-1,-2, and -3

**Scheme 1:**
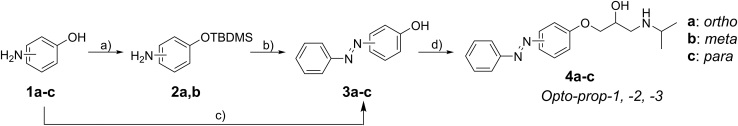
Synthesis of photoswitchable compounds 4a-c (Opto-prop-1, -2, -3) Key: (a) TBDMS-Cl, imidazole, DMF, RT, 3 h – overnight, 99%. (b) [1] PhNO, AcOH, RT, overnight; [2] TBAF, THF, RT, 30 min, 51–70% two steps. (c) PhNO, AcOH, RT, 24 h, 26%. (d) [1] NaOH, EtOH, *rac*-epichlorohydrin, 50 ^°^C, overnight; [2] *i*PrNH_2_, RT, 1 h, 22–41% two steps. Detailed synthesis procedures can be found in Data S1.

Propranolol is a well-known antagonist at both the β_1_-AR and the β_2_-AR (Baker, 2005). In previous work, we successfully changed the naphthalene core of another GPCR ligand to the photoswitchable azobenzene moiety, resulting in only a limited loss in the target-binding affinity of the active isomer. Here we made use of a similar azologization strategy on the naphthalene core of propranolol (Figure 1) (Broichhagen et al., 2015; Hauwert et al., 2018). Thus, compounds Opto-prop-1, Opto-prop-2 and Opto-prop-3 were designed with an azobenzene substituted on the *ortho*, *meta*, and *para* position of the phenyl ring, respectively, with the amino–alcohol recognition element typical for β_2_ antagonists (Chan et al., 2016; Ishchenko et al., 2019). The synthetic sequence is shown in Scheme 1. First, the three regioisomeric asminophenols **1a-c** were, either through the intermediacy of the TBDMS-protected (Petronijevic and Wipf, 2011; Zhang et al., 2013) aminophenols **2a,b** or without protection (Steinwand et al., 2015), to the hydroxyazobenzenes **3a-c** using PhNO. In a one-pot fashion based on classical means (Schwender et al., 1973, 1975), phenols **3a-c** were reacted with *rac*-epichlorohydrin and the intermediate epoxide opened by *i*PrNH_2_ to provide racemic photoswitchable compounds **4a-c**, Opto-prop-1, -2, -3. It should be mentioned that Opto-prop-3 has been reported as a synthetic aniline precursor (Glozman et al., 1987).

**Figure 2:**
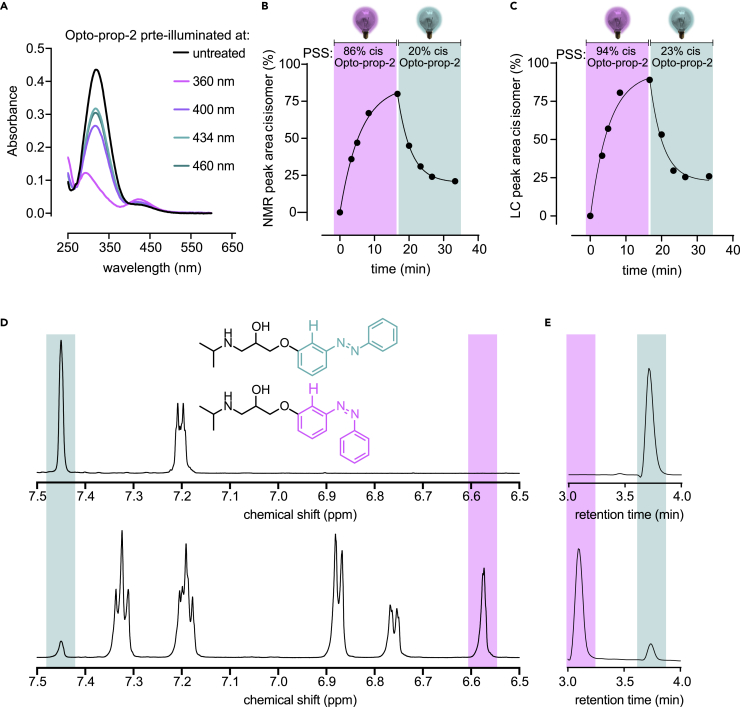
Light-mediated isomer switching of Opto-prop-2 (A) UV-Vis spectroscopy analysis of a sample of *trans*-Opto-prop-2 (25 μM in 1% DMSO/Trs-HCl buffer) after illumination at the indicated wavelengths for 5 min. Panels (B–E) depict the kinetic analysis of Opto-prop-2 isomerization (10 mM in DMSO-d6) upon illumination with 360- and 434-nm light. *Trans*- Opto-prop-2 was illuminated for 1,000 s with 360-nm light and the resulting solution of Opto-prop-2 at PSS_*cis*_ was illuminated for another 1,000 s with 434-nm light. At various time points, a sample was extracted and the amount of *cis*- Opto-prop-2 was determined by (B) the respective NMR peak area or (C) LCMS absorbance peak area at at the isosbestic wavelength (263 nm). (D) NMR-spectrum of Opto-prop-2 before illumination (*t* = 0, upper panel) and after 1,000 s of illuminating with 360-nm light (lower panel). The *x* axis shows only a part of the aromatic region for clarity. The key resonances used for area integration in panel B (analyzing the chemical shift for the hydrogen atom depicted in bold in the structure) are highlighted. (E) LCMS analysis of Opto-prop-2 before illumination (*t* = 0, upper panel) and after 1,000 s of illuminating with 360-nm light (lower panel). Samples were withdrawn, diluted with MeCN and subjected to LCMS analysis at the isosbestic wavelength (263 nm).

Solubility in buffer was explored for all compounds using nephelometry (Bevan and Lloyd, 2000) and these studies indicate good solubility of all azobenzene photoswiches up to at least 100 μM (Figure S1). The absorbance spectra of the azobenzenes were determined in the buffer in the dark and after illumination with either 360 or 434 nm, which are known to induce azobenzene isomerization to the *cis-* and *trans*-isomer, respectively (Hauwert et al., 2018). For all compounds, 360 nm induces a large change in absorbance spectrum compared with the non-illuminated (dark) compounds (Table 1, Figure 2A, and S1), indicating photoisomerization to the *cis*-isomer. The absorbance peak (λ_max_) of Opto-prop-1-3 ranged between 319 and 347 nm for the *trans*-isomers and between 423 and 429 for the *cis*-isomers (Table 1), as is typical for azobenzene compounds (Hauwert et al., 2018). This confirms 360 and 434 nm to be useful illumination wavelengths for *trans-* to *cis-*, and *cis-* to *trans*-switching, respectively. The photochemical conversions were determined by continuous illumination of the Opto-prop compounds with near UV-light (360 ± 20 nm) as 10-mM solutions in DMSO. Based on LCMS analysis, it was observed that all compounds obtained a high level of conversion to the *cis*-isomer with a photostationary state (PSS) of >88 area % *cis*-isomer (i.e. PSS_*cis*_; Table 1). Subsequent back-switching to PSS_*trans*_ with 434 nm maintained 22–29 area % *cis*-isomer (Table 1). The thermal relaxation of the PSS_*cis*_ state of the photoswitchable ligands was monitored over time by measuring absorbance at 254 nm. Only minor thermal relaxation to the *trans*-isomer could be detected at 20°C (not shown). An estimate of the relaxation rate at 20 °C was determined using a method by Priimagi et al. (Ahmed et al., 2017), which was performed in Hanks’ balanced salt solution (HBSS) using increased temperatures to enhance the speed of thermal relaxation. The Opto-prop ligands are all very stable as *cis*-isomer after illumination, with thermal relaxation half-lives of >10 days at 20 °C (See Table 1 and Figure S1).

**Table 1:** Photochemical characterization and receptor binding affinities for Opto-prop-1, -2, and -3

Compound	Chemical characterization					Binding affinity at the β_1_-AR^d^			Binding affinity at the β_2_-AR^d^		
	λ_max__*trans*_^a^ (nm)	λ_max__*cis*_^a^ (nm)	PSS_*cis*_^b^ (% *cis*-isomer)	PSS_*trans*_^b^ (% *cis*-isomer	t_1/2_ 20°C^c^	pK_i_*trans*	pK_i_ PSS_*cis*_^e^	Active state (fold K_i_)^f^	pK_i_*trans*	pK_i_ PSS_*cis*_^e^	Active state (fold K_i_)^f^
**Opto-prop-1**	321	423	88.9 ± 1.0	28.8 ± 0.3	>10 days	8.1 ± 0.1	7.1 ± 0.1	*trans* (9.4)	9.1 ± 0.3	8.5 ± 0.4	*trans* (4.3)
**Opto-prop-2**	319	424	92.8 ± 0.4	25.9 ± 0.4	>10 days	5.3 ± 0.1	6.7 ± 0.0	*cis* (21)	5.8 ± 0.3	8.6 ± 0.3	*cis* (587)
**Opto-prop-3**	347	429	93.8 ± 0.6	21.5 ± 0.1	>10 days	7.9 ± 0.2	6.7 ± 0.1	*trans* (18)	5.0 ± 0.1	5.4 ± 0.1	*cis* (2.6)
**propranolol**	N/A	N/A	N/A	N/A	N/A	9.3 ± 0.1	N/A	N/A	9.8 ± 0.2	N/A	N/A

a Extracted from UV-Vis spectra (25 μM in HBSS buffer with 1% DMSO).

b Measured in DMSO (10 mM) after illumination with 360 nm to PSS_*cis*_ and subsequently with 434 nm to PSS_*trans*_ at room temperature and defined as the percentage of area of the *cis*-isomer compared with combined areas of *cis-* and *trans*-isomers as detected by LCMS analysis with the corresponding isosbestic points wavelength: 278, 263, and 308 nm for Opto-prop-1-3, respectively (*n* = 3, SD given)

c As approximated from a PSS_*cis*_ sample (25 μM in HBSS buffer with 1% DMSO) with the Arrhenius method and extrapolation to the indicated temperature (Ahmed et al., 2017).

d Mean pK_i_ values ±SD are depicted of ≥3 experiments.

e Compounds were illuminated to PSS_*cis*_ before experimentation.

f Fold difference between the K_i_ of the *trans* compared with the K_i_ measured for PSS_*cis*_. N/A = not applicable.

The photochemical properties were examined in more detail for Opto-prop-2 (Figure 2). Opto-prop-2 isomerization was monitored in HBSS buffer with UV-Vis spectroscopy (Figure 2A) after illuminating with various wavelengths, confirming that 360 and 434 nm were most suitable for switching Opto-prop-2 to its *cis*-state (PSS_*cis*_) and *trans*-state (PSS_*trans*_), respectively. The kinetic properties of light-induced isomer switching were further characterized using these wavelengths. Switching was monitored by ^1^H NMR (Figures 2B and 2D) and LCMS (Figures 2C and 2E) analyses in tandem. The ^1^H NMR signal of the *ortho* C-H proton adjacent to the ether function was selected as a clearly resolved signal to quantify the level of isomerization (Figure 2D). The time-dependent isomerization of Opto-prop-2 using 360-nm light has a fitted half-life of 4 min and provides, at PSS_*cis*_, 86 mol% *cis*-isomer. When consecutively isomerizing Opto-prop-2 using 434 nm light, an isomerization half-life of 3 min was observed with 20 mol% *cis*-isomer at PSS_*trans*_ (Figure 2B). Comparable trends were observed in LCMS analysis, with fitted isomerization half-lives of 4 and 3 min to reach PSS_*cis*_ and PSS_*trans*_ with fitted values of 94% and 23% *cis*-isomer, respectively (Figure 2C).

### Opto-prop-2 is a *cis*-active ligand of β_1_-and β_2_-ARs

**Figure 3:**
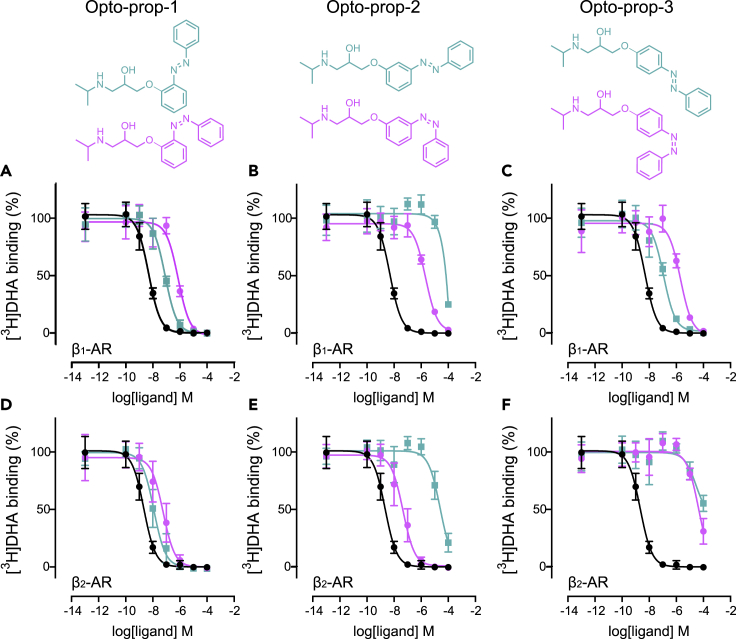
Light-induced modulation of ligand affinity at the β_1_-AR and β_2_-AR Binding of the radioligand [^3^H]DHA to membranes of HEK293T-cells expressing the β_1_-AR (A–C) or the β_2_-AR (D–F) was determined in the presence of increasing concentrations of competing ligands. The competing ligands Opto-prop-1 (A and D), Opto-prop-2 (B and E), and Opto-prop-3 (C and F) were used without prior illumination (*trans*-isomer; green squares) or at PSS_*cis*_ (>94% *cis-*isomer; magenta circles). The reference ligand propranolol (black circles) is depicted in all graphs for comparison. All data points represent the mean ± SD of >3 experiments with individual triplicates.

As all Opto-prop analogs could be efficiently switched to high levels of *cis*-isomer with good thermal stability in an aqueous buffer, the compounds were tested as *trans-* and PSS_*cis*_ states in radioligand binding experiments. Propranolol is known to bind both the β_1_-AR and β_2_-AR with high affinity (Baker, 2005), hence the binding affinities were investigated at both adrenergic receptors (Figure 3). Membranes of HEK293T-cells transiently expressing the receptor of interest were co-incubated with the tritiated β_1_/β_2_-AR antagonist dihydroalprenolol ([^3^H]DHA, Figure S2 depicts saturation binding experiments), together with increasing concentrations of the competing unlabeled ligand. Clear concentration-dependent [^3^H]DHA displacement was observed for Opto-prop-1 (Figures 3A and 3D), Opto-prop-2 (Figures 3B and 3E), and Opto-prop-3 (Figures 3C and 3F) at both the β_1_-AR and the β_2_-AR. Binding affinities (K_i_) of unlabeled ligands were determined from the competition-binding curves by converting the IC_50_ values using the Cheng-Prusoff equation (Table 1).

Interestingly, large differences in the K_i_ values were observed between the *trans-* and the PSS_*cis*_ states of the Opto-prop analogs between *trans-* and *cis*-states of the azobenzenes. At the β_1_-AR the *trans*-isomer of both the *ortho* (Opto-prop-1, Figure 3A) and *para*-substituted azobenzene (Opto-prop-3, Figure 3C) have a high affinity (K_i_ <13 nM), which was markedly decreased upon switching to the *cis*-isomer (9- and 18-fold at PSS_*cis*_, respectively), as observed by a rightward-shift of the inhibition curve. Interestingly, both isomers of Opto-prop-1 selectively bind the β_2_-AR over the β_1_-AR (≥10-fold), whereas both isomers of Opto-prop-3 selectively bind the β_1_-AR over the β_2_-AR (≥10-fold). The β_2_-AR selective ligand Opto-prop-1, however, shows only a modest difference in binding affinity between the isomers (4-fold) at the β_2_-AR (Figure 3D).

In contrast to Opto-prop-1 and -3, which show a reduced binding affinity upon illumination (*trans*-active), the *meta*-substituted azobenzene, Opto-prop-2, shows increased binding affinity after illumination (Figures 3B and 3E), resulting in a 21-fold increase in binding affinity at the β_1_-AR and a striking 587-fold increase in affinity at the β_2_-AR. Next to the excellent *cis*-active photomodulatory properties at the β_2_-AR, the *cis*-isomer of Opto-prop-2 also has high selectivity in binding the β_2_-AR over the β_1_-AR (78-fold). Consequently, Opto-prop-2 represents a unique *cis*-active β_2_-AR antagonist photoswitch with a large window of optical modulation and long thermostability. Opto-prop-2 was therefore selected for further biological characterization.

### Opto-prop-2 has photoswitchable betablocker activity

**Figure 4:**
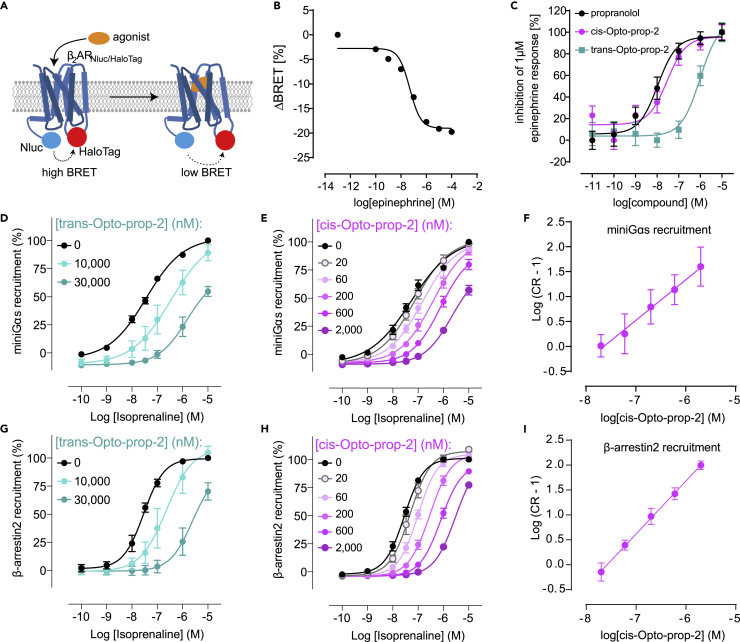
Light-dependent effects of Opto-prop-2 on β_2_-AR activity in cell-based experiments Conformational changes of the β_2_-AR can be monitored using an intramolecular BRET sensor (A). Upon binding of an agonist, the β_2_-AR adopts an active conformation in which the distance between the BRET donor Nluc and BRET acceptor (NanoBRET-618-labeled HaloTag) is enlarged, resulting in a reduced BRET signal. Conformational changes of the β_2_-AR were determined when incubating cells with epinephrine alone (B) or in the presence of the ligands (C) propranolol (black circles), *trans*-Opto-prop-2 (teal squares) or Opto-prop-2 at PSS_*cis*_ (>94% *cis*; magenta circles). The agonist-induced recruitment of intracellular signaling molecules to the β_2_-AR was determined by nanoluc luciferase-complementation proximity sensors (D–I). The LgBit-fused β_2_-AR recruits the SmBit-fused signaling molecules mini-Gαs (D–F) or β-arrestin2 (G–I) upon stimulation with an agonist. Recruitment for both the mini-Gαs (D and E) and β-arrestin2 (G and H) were determined for increasing concentrations of the agonist isoprenaline in the absence (black circles) or presence of increasing concentrations *trans*-Opto-prop-2 (D and G) or Opto-prop-2 at PSS_*cis*_ (>94% *cis*; E and H). Schild-analysis of the *cis*-Opto-prop-2 induced dextral shifts of the isoprenaline concentration–response curve are depicted for mini-Gαs recruitment (F) and β-arrestin2 recruitment (I) All data points represent the mean ± SD of three experiments.

Using a conformational biosensor of β_2_-AR, we sought to investigate whether Opto-prop-2 can inactivate the agonist-bound receptors on living cells and to what extent this could be regulated with light. The conformational β_2_-AR sensor allows detection of the active receptor conformation using a bioluminescence resonance energy transfer (BRET)-readout (Figure 4A) (Schihada et al., 2018). When HEK293A cells that stably express this conformational sensor are activated by epinephrine, a clear concentration-dependent decrease in BRET was observed (pEC_50_ = 7.3 ± 0.2), as is typical for agonist-mediated activation of the β_2_-AR sensor (Figure 4B) (Schihada et al., 2018). This epinephrine-induced effect could be inhibited by *trans*-Opto-prop-2, but only at high concentrations (pIC_50_ = 6.0 ± 0.2, Figure 4C). When Opto-prop-2 was activated with light, however, the inhibitory potency was markedly increased (pIC_50_ = 7.5 ± 0.2), as shown by the leftward shift of the inhibition curve (Figure 4C). The inhibitory effect of Opto-prop-2 on the agonist-induced conformational change suggests antagonism at β_2_-AR, similar to the structurally related propranolol (pIC_50_ = 8.0 ± 0.2). The pK_B_ of Opto-prop-2 calculated from its inhibitory effect on the β_2_-AR conformational sensor (8.7) was similar to the affinity determined in radioligand binding experiments (Table 1).

To confirm that Opto-prop-2 antagonizes β_2_-AR signaling in a light-dependent way, two enzyme complementation assays were used to measure the intracellular recruitment of mini-Gα_s_ (black graph; Figures 4D–4F) and β-arrestin2 (black graph; Figures 4G–4I) to β_2_-AR in response to the agonist isoprenaline (Carpenter and Tate, 2016; Dijon et al., 2020; Nehmé et al., 2017). In the presence of Opto-prop-2, the concentration-response curve of isoprenaline shifts to the right, as is expected for competitive antagonism. When comparing the dextral shifts of the isoprenaline concentration-response-curves in the presence of *trans*-Opto-prop-2 (Figures 4E and 4H) with the illuminated *cis-*Opto-prop-2 (Figures 4F and 4I), it is clear that 360-nm light potentiates the antagonism of Opto-prop-2, as considerably higher concentrations of *trans*-Opto-prop-2 are needed to shift the isoprenaline concentration-response-curves. Schild analysis (Arunlakshana and Schild, 1959) (Figures 4F and 4I) of Opto-prop-2 at PSS_*cis*_ indeed confirms competitive antagonism of isoprenaline-induced β_2_-AR activation, with a slope of the Schild-plot of 1.2 and 0.9 and a pA_2_ of 7.6 and 7.6, for mini-Gα_s_ (Figure 4F) and β-arrestin2 (Figure 4I) recruitment, respectively.

**Figure 5:**
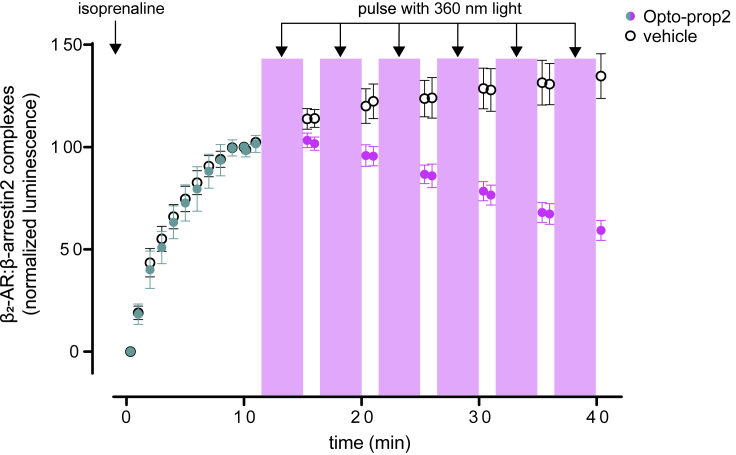
Temporal regulation of β_2_-AR activity using Opto-prop-2 HEK293T-cells transiently expressing β_2_-AR-SmBit and βarrestin2-LgBit were stimulated at time point 0 using 32 nM isoprenaline with or without Opto-prop-2 (1 μM) as depicted by closed and open circles, respectively. β-arrestin2 binding to β_2_-AR was monitored by measuring the luminsecence for 10 min after which cells were pulsed with 360 nm for six cycles of 5 min with an intermittent readout of luminescence. Cells incubated with Opto-prop-2 are depicted in green and magenta before and after illumination, respectively. Data points represent the mean ± SEM of the three experiments, normalized to the luminescence at 10 min as 100%.

It was further explored whether the activity of the β_2_-AR could be modulated dynamically by treating cells with light (Figure 5). Cells that overexpress a split-nanoluc luciferase biosensor system for βarrestin2 recruitment to the β_2_-AR were treated with isoprenaline in the presence of Opto-prop-2. A time-dependent activation of the receptor is observed for 10 min after which cells were pulsed with light flashes of 360 nm. After each cycle with low-energy light flashes (depicted with magenta box), a drop in β_2_-AR activity is observed for the cells that were treated with Opto-prop-2.

**Figure 6:**
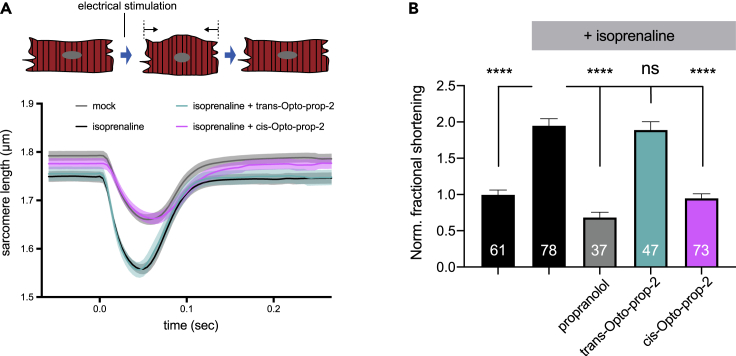
Light-dependent betablocker activity of Opto-prop-2 on freshly isolated adult rat cardiomyocytes Electrical pacing of rat cardiomyocytes resulted in transient shortening of the sarcomeres, which was monitored over time as depicted in panel (A) for cells treated with vehicle (DMSO, gray line), 15-nM isoprenaline (black line), or isoprenaline in combination with *trans*-Opto-prop-2 (teal line) or *cis*-Opto-prop-2 (PSS_*cis*_; magenta line) at 1 μM. A representative graph is depicted, with the average sarcomere length (line) and SEM (shading) of cardiomyocytes measured on the same day. In panel (B), the normalized maximal shortening of cardiomyocytes is depicted for each perturbation with the mean ± SEM The number of replicate measurements is depicted per condition in the respective bar graph. Bar graphs were compared by one-way ANOVA with Dunnett’s multiple comparison. ns = no significant difference; ∗∗∗∗significant difference with *p* < 0.001.

To evaluate Opto-prop-2 in a relevant biological system with endogenous levels of the native receptor proteins, it was tested using isolated adult rat cardiomyocytes. Cardiomyocytes were paced at 2Hz, inducing contractions that were quantified by measuring the sarcomere length over time (Figure 6A). The β_2_-AR agonist isoprenaline (15 nM) further increased the shortening of the sarcomeres (black line) (Najafi et al., 2016a). This enhanced contractility was attenuated by co-incubating with *cis*-Opto-prop-2 (magenta line) but not by *trans*-Opto-prop-2 (teal line) at 1 μM concentrations. The normalized peak contraction is depicted for each perturbation (Figure 6B), showing that antagonism of isoprenaline by Opto-prop-2 is fully dependent on its light-induced isomerization to PSS_*cis*_, after which a similar level of antagonism is observed for the reference beta-blocker propranolol.

## Discussion

Spatiotemporal control of drug action by light is potentially a new way forward toward effective localized treatment with limited on-target side effects (Welleman et al., 2020; Wijtmans et al., 2022). The optical modulation of GPCRs with photoswitchable ligands has recently received considerable interest (Berizzi and Goudet, 2020; Ricart-Ortega et al., 2019; Wijtmans et al., 2022) in view of the importance of GPCR-based drugs in today’s pharmacy (Santos et al., 2017). In this study, we have focused on the development of photoresponsive propranolol analogs. Propranolol is a hall-mark beta-blocker, that effectively antagonizes both β_1_-AR and β_2_-AR at nanomolar concentrations (Baker, 2005). Moreover, the drug is effectively used to treat cardiovascular diseases (Freemantle et al., 1999; Zhang et al., 2017). Also, beta blockers such as propranolol are used to treat infantile hemangiomas (Al-Haddad et al., 2019; Hagen et al., 2018), although data on observed side effects are questioning the route of administration and advocating locally acting drugs. Consequently, photoresponsive propranolol analogs might be a good addition to the pharmacological toolbox to study mechanistic aspects of spatiotemporal beta-adrenergic receptor signaling, but ultimately even become of therapeutic use.

In this study, we designed and synthesized three photoswitchable propranolol analogs by replacing the naphthalene moiety in propranolol with the photochromic azobenzene unit and linking this with the propranolol sidechain at either the *ortho*, *meta*, or *para* position (Figure 1). All analogs can be effectively photoswitched (PSS_*cis*_ ≥ 94%) by illumination at 360 nm. Moreover, the half-lives of the thermal backswitching of the *cis*-isomers are all very long (>33 h, Table 1), making them suitable for most assay formats.

The Opto-prop-1, -2, and -3 ligands were characterized in detail by [^3^H]-DHA radioligand binding studies with both the human β_1_-AR and β_2_-AR, heterologously expressed in HEK293T-cells. Remarkable differences in β-AR pharmacology were observed. For example, an 813-fold selectivity in the binding affinity to β_1_-AR over β_2_-AR was observed for the active isomer of *trans*-Opto-prop-3, whereas a 21-fold and 78-fold selectivity in favor of the β_2_-AR were observed for the *cis*-Opto-prop-1 and *cis*-Opto-prop-2, respectively (Table 1). Such differences in selectivity are not entirely unexpected, as it has been shown that substituting the aromatic region of the class of phenoxy-propanolamines can affect the binding selectivity at the β_1_/β_2_ adrenergic receptors (Labrid et al., 1989; Ruffolo et al., 1995).

Opto-prop-1, -2, and -3 all show large differences in binding profiles between their *trans-* and *cis*-isomers at the β_1_/β_2_ adrenergic receptors, albeit to a different extent and dependent on the actual adrenergic receptor subtype. At the β_1_-AR, both Opto-prop-1 and -3 are *tran*s-active photoswitches, leading to a 9- to 18-fold loss in affinity, respectively after 360 nm illumination. In contrast, the *meta*-substutited analog Opto-prop-2 is a *cis*-active compound at β_1_-AR, but only binds at (sub)micromolar concentrations. At the β_2_-AR, both Opto-prop-1 and -2 show interesting properties, with Opto-prop-1 being a high-affinity binder with only a slight (4.3-fold) loss in affinity. Yet, the *trans*-isomer of Opto-prop-2 binds β_2_-AR at micromolar concentrations, but shows substantial light-enhanced binding affinity after illumination with 360 nm. The increase in affinity of almost 3 log-units for the *cis*-Opto-prop-2 makes *cis*-active Opto-prop-2 one of the photoswitches with the highest affinity and the largest optically-induced shift in affinity for family A GPCRs (Wijtmans et al., 2022). The nanomolar affinity of the *cis*-isomer also makes Opto-prop-2 the best β_2_-AR-specific compound of the series with a 1.9-log unit difference in binding affinity with β_1_-AR (Table 1). In general, the practical utility of *trans*-active ligands (such as Opto-prop-1 and Opto-prop-3) is restricted by the inability to reach high conversion to the *cis*-isomer upon illumination, resulting in residual effects of the more active *trans-*isomer (Wijtmans et al., 2022). Therefore, *cis*-active ligands are generally preferred, as the light-induced shift in biological activity can be much higher as clearly demonstrated by Opto-prop-2 with a 587-fold increase in β_2_-AR binding affinity upon illumination (Table 1). Whereas *cis*-on compounds Bode well for appreciable differences in biological activity when switching from *trans* to *cis*, dynamic optical control of GPCR activity will be critically depending on the amount of *cis*-isomer left at PSS_*trans*_. With classical azobenzenes, such PSS_*trans*_ values are typically relatively high, as is the case for Opto-prop-2 (26% *cis*-isomer at PSS_*trans*_). Consequently, Opto-prop-2 maintains a substantial affinity at PSS_*trans*_ (pK_i_ = 7.5 ± 0.1, compared with pK_i_ = 8.6 ± 0.3 for PSS_*cis*_).

Based on the *cis*-active nature, the β_2_-AR selectivity, and the nanomolar affinity of the *cis*-isomer, Opto-prop-2 was selected for more detailed studies at the β_2_-AR. Using an established BRET-based β_2_-AR conformational sensor (Schihada et al., 2018) and nanobit-based mini-Gα_s_- and βarrestin2-recruitment assays for β_2_-AR (Dijon et al., 2020, 2021), both isomers of Opto-prop-2 proved to be β_2_-AR antagonist, with a clear *cis*-active profile, as observed in the radioligand binding studies. Moreover, using the option to illuminate cells within the microplate reader with consecutive, low-energy 360 nm flashes, also real-time Opto-prop-2 switching and antagonism of β_2_-AR function could be shown, highlighting the usefulness of Opto-prop-2 for future photopharmacology applications.

As shown, Opto-prop-2 binds both β_1_-AR and β_2_-AR upon *trans-cis* isomerization, but with higher selectivity for the β_2_-AR than propranolol (Table 1). It was therefore explored whether the specific profile of Opto-prop-2 can be used for light-induced inhibition of rat cardiomyocytes contractility, as these cells are known to express both β_1_-AR and β_2_-AR subtypes (Devic et al., 2001; Najafi et al., 2016b). As shown in this study, Opto-prop-2 also functions as a photoswitchable beta-blocker (Figure 6) in cardiomyocytes that endogenously express both the β_1_ and β_2_ adrenergic receptors.

Recently, photoswitchable β_2_-AR antagonists similar to the current Opto-prop designs have been published by Duran-Corbera et al. (2020) In this work, which uses an approach that we had been exploring in parallel (Bosma, 2019; Leurs, 2020), an azologization strategy of propranolol included the decoration of the posterior phenyl ring of the azobenzene with a *para*-acetamido group. Compared with the Opto-prop ligands, these *para*-acetamido analogues have a much faster thermal relaxation time (73–169 min at 25 °C) and a small shift in peak absorbance wavelengths. These changes in photochemical properties are consistent with prior work, in which increasing the electron density of azobenzenes is shown to increase the rate of thermal relaxation and red-shifting of the absorbance spectrum (Bandara and Burdette, 2012; Küllmer et al., 2022). The levels of *cis*-isomer at PSS_*cis*_ and PSS_*trans*_ seem to be similar upon substituting with the *para*-acetamido moiety. A slight improvement in the *trans*-content was seen at PSS_*trans*_ for the substituted Opto-prop3 (from 78.5% to 86.2%), but Duran-Corbera et al. did not exploit this, owing to its relative inactivity at the β_2_-AR. Interestingly, substituting Opto-prop-1 with the *p*-acetamido substituent results in a slightly larger optically-induced shift in antagonist potency at β_2_-AR compared with the non-substituted azobenzene Opto-prop-1. With a 17-fold reduction in β_2_-AR blockade upon illumination (*trans*-active), this *para*-acetamido analog had the largest light-induced shift in antagonistic potency of their series. However, substituting Opto-prop-2 with a *para*-acetamido moiety was clearly not ideal for photoswitching at β_2_-AR. The observed 3.6-fold difference in β_2_-AR antagonistic potency in favor of the *cis*-isomer (Duran-Corbera et al., 2020) is striking in view of the 587-fold difference observed in this study for the *trans*- and *cis*-isomers of Opto-prop-2. This comparison suggests that the *meta-*substituted azobenzene binds quite well to the β_2_-AR binding pocket in its *cis*-configuration, but can not easily accommodate an additional *para*-acetamido substituent. Future molecular modeling, combined with site-directed mutagenesis studies and/or structural biology studies will be needed to resolve the intricate details of the binding of this class of photoswitches to the β_2_-AR. Similarly, such studies might also shed light on the observed β_1_-AR/β_2_-AR selectivity of Opto-prop-1, -2, and -3.

In conclusion, the low-affinity photoswitch *trans*-Opto-prop-2 (VUF17062) is shown to efficiently switch to its high-affinity *cis*-isomer (587-fold increase) under the influence of 360-nm light, resulting in a nanomolar affinity at the β_2_-AR and selectivity over the β_1_-AR. The high β_2_-AR affinity of *cis*-Opto-prop-2 and its large photo-induced shift in affinity, make Opto-prop-2 one of the exceptional useful photoswitches for family A GPCRs (Wijtmans et al., 2022). Its *cis*-active nature, photochemical and photopharmacological properties make Opto-prop-2 a promising chemical biology tool for the study of spatiotemporal β_2_-AR signaling in various biological contexts. Moreover, further development of Opto-prop-2 analogs toward more red-shifted analogs would ultimately allow the use of wavelengths in the visible spectrum, making them more compatible with *in vivo* use (Wijtmans et al., 2022).

### Limitations of the study

Opto-prop-2 is an effective tool to optically control the activity of the β_2_-AR. Future optimization could focus on the percentage isomer switching, in particular at PSS_*trans*_. This could further increase the dynamic switching between active and inactive β_2_-AR. Moreover, it might be possible to improve selectivity for the β_2_-AR over the β_1_-AR. Although the 78-fold difference in affinity is already substantial, larger differences would allow more stringent control of the β_2_-AR (i.e., at higher concentrations) without affecting the β_1_-AR.

## STAR★Methods

### Key resources table

**Table:** 

REAGENT or RESOURCE	SOURCE	IDENTIFIER
**Chemicals, peptides, and recombinant proteins**		

Opto-prop-1	This paper	See supplemental information
Opto-prop-2	This paper	See supplemental information
Opto-prop-3	This paper	See supplemental information
Propranolol	In-house library	CAS 525-66-6
Isoprenaline	Tocris	Cat. 1747; CAS 7683-59-2
(−)epinephrine	Sigma Aldrich	Cat. E4250; CAS 51-43-4
[^3^H]dihydroalprenolol	Perkin Elmer	Cat. NET720
Liberase TM research grade	Roche	LIBTM-RO

**Critical commercial assays**		

Lipofectamine 3000	Invitrogen	Cat. L3000001
25 kDa polyethyleneimine	Polysciences	Cat. 23966, CAS 9002-98-6
HaloTag dye NanoBRET 618	Promega	Cat. G9801
NanoGlo (furimazine)	Promega	Cat. N1130
Microscint-O	Perkin Elmer	Cat. 6,013,611

**Experimental models: Cell lines**		

HEK293A	Thermo Fischer	Cat. R70507
HEK293T	ATCC	Cat. CRL-1573

**Experimental models: Organisms/strains**		

Wistar rats	Charles River	N/A

**Recombinant DNA**		

pcDEF_3_ - β_1_AR	Biomatik	Custom synthesis
pcDNA3.1(+) - β_2_AR	cDNA resource center	Cat. AR0B200000
pcDNA3 - β_2_AR_Nluc/HaloTag_	Schihada et al., (2020) (54)	N/A
pcDEF_3_ - β_2_AR-SmBit	This paper	N/A
pBiT1.1-C - βarrestin2-LgBit	Ma et al., (2021) (53)	N/A
pcDNA3.1(+) - SNAP-hβ_2_AR-LgBit	Dijon et al. (36)/this paper	N/A
pcDNA3.1zeo(+) - SmBit-Gα_s_	Dijon et al. (36)/this paper	N/A
pcDNA3.1zeo(+) - SmBit-βarrestin2	Dijon et al. (36)/this paper	N/A

**Software and algorithms**		

GraphPad Prism 8	GraphPad Software	www.graphpad.com
Ionoptix software	Ionoptix	www.ionoptix.com
Transient Analysis Tools	CytoCypher	www.ionoptix.com

### Resource availability

#### Lead contact

Requests for materials and information can be addressed to the Lead Contact, Rob Leurs, r.leurs@vu.nl.

#### Materials availability

Plasmids generated in this study will be made available upon reasonable requests.

### Experimental model and subject details

In the current research the female cellines HEK293T and HEK293A and the primary cardiomyocytes isolated from 6-8 week old male Wistar rats. Cells are cultured at 37 °C in a humidified atmosphere with 5% CO_2_. Further details are described in the method details.

### Method details

#### Materials

##### Constructs

The codon-optimized human β_1_AR in pcDEF_3_ (NCBI: NP_000675.1) in pcDEF_3_ was synthesized by Biomatik (the Netherlands) and human β_2_AR in pcDNA3.1+ was purchased from the cDNA resource center (USA) which corresponds to GenBank accession NM_000024.3. The mini-Gαs gene sequence synthesis (Carpenter and Tate, 2016; Nehmé et al., 2017) (GeneArt) was purchased from Invitrogen (Paisley, UK) and Nanoluciferase constructs (Dixon et al., 2016) (LgBiT, SmBiT NanoBiT fragments) were obtained from Promega corporation (WI, USA). The codon-optimized sequence of human β_2_AR was synthesized in frame with SmBit spaced by a Gly/Ser linker (Ma et al., 2021) and was cloned in a pcDEF_3_ vector. βarrestin2-LgBit was described previously (Ma et al., 2021). The β_2_AR_Nluc/HaloTag_ contains the cDNA of the β_2_AR with the HaloTag in the third intracellular loop between Asp251 and Gly252 and Nluc in the C-terminus at Glu369 in pcDNA3, as described previously. (Schihada et al., 2020) The SNAP-h β_2_AR-LgBit is based on the hβ_2_AR cDNA (GenBank: NM_000024.6) modified with an N-terminal SNAP tag (New England Biolabs, Hitchen UK) and the LgBiT sequence with Leu-Glu linker (Dixon et al., 2016) at the C terminus, in pcDNA3.1(+). βarrestin2-SmBit and mini Gα_s_-SmBit plasmid DNA contain the cDNA of βarrestin2 (GeneBank: NM_004313) or mini Gα_s_ with an N terminal SmBiT sequence followed by 5 Ser/Gly linker, in pcDNA3.1 zeo (+).

##### Compound use in biological experiments

The HaloTag dye NanoBRET 618 and stock solution furimazine (Nano-Glo) were obtained from Promega. Isoprenaline was obtained from Tocris (UK) and (−)epinephrine was purchased from Sigma Aldrich (USA). Propranolol was obtained from an in-house library and was confirmed to be of analytical purity. Opto-prop-1 (VUF17061), Opto-prop-2 (VUF17062) and Opto-prop-3 (VUF25417) were synthesized and compounds were analyzed with respect to their identity and purity as described in the supplemental information (Data S1).

#### Nephelometry measurement

In transparent flat-bottom 96-well plates, the azobenzene propranolol analogs, under dark conditions or preilluminated with 360 nm light to PPS_*cis*_, were placed at different concentrations in triplicate (10^−4^ M, 10^−4.5^ M, 10^−5^ M, 10^−5.5^ M, 10^−6^ M, 10^−6.5^ M, 10^−7^ M, 10^−7.5^ M and a blank) in aqueous buffer with 1% DMSO at least 1 h before the measurement. A Kaolin dispersion was used as a positive control.(Roessler and Brewer, 1967) Nephelometry measurements were performed with a NEPHELO star Plus (BMG Labtech, Germany) with the following settings: laser intensity 80%, beam focus 2.0 mm, and Orbital shaking of 10 s at 200 rpm before data acquisition. Results were analyzed using GraphPad Prism 8 software, plotting all available data points and plotting mean and SD values in a line chart compared to kaolin control. The linear fit (R^2^) of the kaolin control was above 0.99 in all cases.

#### Photochemistry procedures

UV-Vis spectra were obtained using a Thermo-scientific Evolution 201 PC spectrophotometer. Illumination was executed using a Sutter instruments Lambda LS with a 300 Watt full-spectrum lamp connected to a Sutter instruments Lambda 10-3 optical filter changer equipped with 360 ± 20 nm, 400 ± 5 nm, 434 ± 9 nm and 460 ± 5 nm filters. The light intensity is 0.93 mW/mm^2^ using the 360 ± 20 nm filter, 0.22 mW/mm^2^ using the 400 ± 5 nm filter, 0.79 mW/mm^2^ for the 434 ± 9 nm filter and 0.26 mW/mm^2^ for the 460 ± 5 nm filter as measured using a Thorlabs PM16-401 power meter.

For the determination of the UV-Vis spectra shown in Figure 2A, compounds (25 μM in 1% DMSO/HBSS buffer) were illuminated in Hellma Suprasil quartz 114-QS cuvettes with either a 360, 400, 434 or 460 nm filter for the indicated time. For the determination of PSS_*cis*_ and PSS_*trans*_ states by UV spectroscopy (Figures S1D–S1F), a solution of the compound in 1% DMSO/HBSS buffer (25 μM) in a vial was illuminated to PSS *cis* with 360 nm (typically 4–5 min) and subsequently to PSS *trans* with 434 nm (typically 2–5 min). UV spectra were recorded.

For the determination of PSS_*cis*_ and PSS_*trans*_ values by LC analysis (Table 1), a solution of the compound in DMSO (10 mM) was illuminated to PSS_*cis*_ with 360 nm and subsequently to PSS_*trans*_ with 434 nm. PSS aliquots were withdrawn, diluted with MeCN and subjected to LCMS analysis with the corresponding isosbestic points wavelength: 278, 263 and 308 nm for Opto-prop-1-3, respectively.

*Cis*-to-*trans* thermal relaxations were performed in the dark at 25 μM of the PSS_360_ state of the Opto-prop-1, -2 or -3 in HBSS buffer with 1% DMSO at various temperatures (60°C, 70°C and 80°C). Absorbance was repeatedly measured at 320, 320 and 347 nm for Opto-prop-1, -2 or -3, respectively, with 60 s intervals. Using an Arrhenius extrapolation method (Ahmed et al., 2017), the relaxation half-life at 20°C was calculated. The linear fit for the Arrhenius equation had an R^2^ of >0.95 in all cases.

For time-resolved detection of Opto-prop-2 isomerization, illuminations were performed a 10 mM sample in DMSO-d_6_ at a volume of 1 mL during 1000 s with a 360 nm or 434 nm filter. Aliquots were withdrawn at NMR measurement times, diluted with MeCN and subjected to LCMS analysis at the isosbestic wavelength (263 nm).

Illuminations for pharmacological experiments were performed at room temperature in cylindrical clear glass vials with a volume of 150 μL during 10 min with a 360 nm or 434 nm filter. Samples were 10 mM in DMSO. The photoisomerization and photostability was monitored by LCMS for all the samples. The typical distance between light source and vial or cuvette was 2 cm.

#### Radioligand binding experiments

##### Membrane production

Two million HEK293T cells were seeded in a 10cm^2^ dish and were transiently transfected the next day with 1 μg human β_1_-AR or the β_2_-AR plasmid DNA, 4 μg empty pcDEF_3_ plasmid DNA and 30 μg linear 25 kDa polyethyleneimine (PEI; Polysciences Warrington, USA) in a 150 mM NaCl solution. (Bosma et al., 2016) Briefly, 250 μL solutions of containing either DNA or PEI in 150 mM NaCL were combined and vortexed. After 15 min the DNA/PEI solution was supplemented to HEK293T cells in fresh culturing medium. Two days after transfection, cells were resuspended and washed (3 times) with PBS and pelleted by centrifugation steps at 1500 g. After the final centrifugation step, cell pellets were reconstituted into ice-cold Tris-HCl buffer (15 mM, supplemented with 0.3 mM EDTA and 2 mM MgCl_2_ at pH7.4) and dounce-homogenised by plunging the pestle 10 times with 1,500 rpm (Tamson, the Netherlands). The homogenates were frozen and thawed twice using liquid nitrogen, after which the membranes were isolated using an ultra-centrifuge L70 at 40,000 g (Beckman Coulter, USA). Cell pellets were then washed and reconstituted in ice-cold 20 mM Tris-HCl buffer supplemented with 250 mM Sucrose at pH 7.4. Finally, the membrane samples where homogenized using a 23-gauge needle, snap-frozen with liquid nitrogen and stored until further experimentation at −80 °C. The protein content was determined using a BCA protein assay kit (Thermo Scientific, USA).

##### Radioligand displacement

On the day of the experiment 0.25–0.5 μg membranes expressing either the β_1_-AR or the β_2_-AR, were co-incubated with [^3^H]DHA (Perkin Elmer, USA) and increasing concentrations unlabeled ligand in HBSS buffer (Thermo Scientific, with Ca^2+^ and Mg^2+^) supplemented with 0.1% BSA for 1 h at 25 °C under gentile agitation. Either a 4 nM or 1 nM concentration of [^3^H]DHA was used in combination with membranes expressing either the β_1_-AR or β_2_-AR, respectively. Binding reactions were terminated by filtration over a PEI coated GF/C filter (Perkin Elmer) using ice-cold 50 mM Tris-HCl buffer with 500 mM NaCl at pH 7.4. GF/C plates were dried at 52 °C and filter bound [^3^H]DHA was quantified by adding 25 μL/well Microscint-O scintillation liquid and counting luminescence using the Wallac Microbeta counter, as described before. (Bosma et al., 2016).

#### Conformational BRET sensor

##### BRET based detection conformational dynamics

HEK293A cells stably expressing β_2_AR_Nluc/HaloTag_ were seeded onto PDL-pre-coated, white 96-well plates (50,000 cells/well) and incubated over night with 50 nM HaloTag dye NanoBRET 618. The next day, cells were washed with HBSS and incubated with a 1/1000 stock solution of furimazine. 5 min later, baseline BRET was recorded in three consecutive reads (4.7 min), 10-fold serial dilutions of epinephrine were added, and the ligand-induced BRET ratio was recorded in 17 consecutive reads (40 min). For competition experiments with propranolol and Opto-prop-2, β_2_AR_Nluc/HaloTag_ expressing cells were incubated with the 1/1,000 stock solution of furimazine along with the indicated concentrations of propranolol, Opto-prop-2 isomers or 0.1% DMSO in HBSS (antagonist vehicle control). After recording the basal BRET ratio, all wells pre-treated with propranolol or Opto-prop-2 were stimulated with 1 μM epinephrine. Wells pre-incubated with 0.1% DMSO/HBSS control were treated with vehicle control. All experiments were conducted using a CLARIOstar plate reader (BMG Labtech) recording Nluc and HaloTag NanoBret 618 emission intensities with 460/40 nm and 620/20 nm monochromator settings, respectively.

#### Nanobit complementation experiments

##### Molecular biology/cell culture

HEK 293T cells (ATCC CRL-1573) were sequentially transfected using Lipofectamine 3000 (Invitrogen, US) in Opti-MEM media (Sigma Aldrich) with SNAP-β_2_AR2-LgBit and either human β-arrestin2-SmBit or mini-Gα_s_-SmBit. Mixed stable populations of HEK-β_2_AR-β-arrestin2, or HEK-β_2_AR-mG_s_ cells were generated by selection in G418 (0.8 mg^−1^) and zeocin (200 μg mL^−1^) for 7–10 days, in DMEM, supplemented with 10% fetal bovine serum (FBS; Sigma Aldrich). For NanoBiT luminescence assays, cells were passaged using trypsin at 70–80% confluency and plated on poly-D-lysine (Sigma Aldrich) coated 96-well flat-bottomed white plates (Greiner 655,098), with 40,000 cells per well.

##### NanoBiT luminescence assays

24 h following plating, NanoBiT assays were conducted in HEPES balanced salt solution (147 mM NaCl, 24 mM KCl, 1.3 mM CaCl_2_, 1 mM MgSO_4_, 1 mM Na pyruvate, 1 mM NaHCO_3_, 10 mM HEPES, pH 7.4, and sterilized by autoclave. D-Glucose added to 10 mM before first use and 0.1% BSA added on the day of experiment). Cells were washed and pre-incubated with buffer or Opto-prop-2 isomers (10 min; 37 °C), followed by furimazine incubation (final assay dilution ratio 1:660, from furimazine stock concentration) for 5 min at 37 °C. Following 3 baseline measures of luminescence read at 2 min intervals, isoprenaline was added and the resulting luminescence was monitored every 2 min for 31 min on a PHERAstar platereader set to 37 °C (BMG Labtech).

##### Kinetic analysis

HEK293T cells were transiently transfected with 2 μg plasmid DNA per million using linear PEI at a DNA:PEI weight-ratio of 1:6. (Zarca et al., 2021) The used DNA plasmids were β_2_-AR-SmBit (0.4 μg), β-arrestin2-LgBit (0.6 μg) and pcDEF_3_ (non-coding DNA; 1 μg). The DNA/PEI transfection mix in 150 mM NaCL was vortexed and incubated for 15 min before adding it to a cell suspension with a final concentration of 300,000 cell/mL. Cells were then immediately plated 30,000 cells/well on a white 96-well plate (Greiner). Cells were then treated with NanoGlo substrate (final assay dilution ration 1:300), 1 μM Opto-prop-2 and 32 nM isoprenaline followed directly by luminescent detection at the CLARIOstar platereader for 10 min at 25 °C. After 10 min the protocol setting were switched to treat cells repeadetly with 360 nm light flashes (40 times 200 flashes over 5 min) followed after each 5 min cycle with detection of luminescence, up to a total detection time of 40 min.

#### Intact cardiomyocyte isolation and measurements

Intact adult cardiomyocytes were isolated from 3 adult wild-type Wistar rats weighing 200–250 g as described previously. (Nollet et al., 2020; van Deel et al., 2017) Briefly, rats were sedated and anesthesized by isoflurane inhalation. The heart was removed and perfused in a Langendorff setup with Liberase TM (Roche) digestive enzymes and the left ventricle was consequently cut into small pieces and resuspended in stopping buffer. This mixture was passed over a 300 μm nylon mesh filter, isolated cells in the filtrate were resuspended in buffer containing 1mM CaCl_2_. Cells were suspended in plating medium composed of Medium 199 (Lonza), 1% penicillin/streptomycin and 5% bovine serum and plated on a laminin coated dish (10 μg/mL, Sigma-Aldrich). The cells were incubated for 1 h at 37 °C in humidified air with 5% CO_2_ to let them attach to the coated dish. Afterwards, non-attached cells were removed by washing cells with pre-heated Tyrode solution (137 mM NaCl, 5.4 mM KCl, 3 mM sodium pyruvate, 5 mM HEPES, 0.57 mM MgCl_2_, 0.33 mM NaH_2_PO_4_, 1.0 mM CaCl_2_ and 5.6 mM glucose, pH 7.4 at 37 °C). Contractility measurements were performed at 37 °C using the Multi-Cell system (CytoCypher, the Netherlands). Cells were treated to vehicle (DMSO), 15 nM isoproterenol alone or in combination with 1 μM of propranolol or Opto-prop-2 (either with or without prior illumination). The dish was field-stimulated at 2 Hz, 25 V and a 4 ms pulse duration.

### Quantification and statistical analysis

#### Photochemical characterization

NMR, LC and UV-Vis spectra were plotted in GraphPad Prism 8 and were visualized by fitting the data to a second order polynomial with a smooth function, using 4 neighboring data points. The aromatic region of the NMR-spectrum is depicted between ppm 6.5 and 7.5 (Figure 2D), with 6,545 intermittent datapoints. For the time-resolved isomerization experiments, integration of the peak areas was performed with Shimadzu and MestreNova software for LC and NMR integration, respectively. The normalized peak areas over time were fitted by non-linear regression in GraphPad Prism 8 using a one-phase exponential model which yields the t_1/2_ value of the isomerization reaction and the extrapolated PSS (i.e. the asymptote).

#### Radioligand binding experiments

IC_50_ values were determined by fitting the concentration-dependent displacement of [^3^H]DHA by unlabeled ligands, with a three-parameter sigmoidal model in GraphPad Prism. The K_i_ of all unlabeled ligands were calculated according to the Cheng-Prusoff equation (Cheng and Prusoff, 1973) using the radioligand binding affinity values determined by saturation binding. Competition binding graphs represent the pooled data of ≥3 experiments, normalized to the fitted top and bottom of the reference sigmoidal displacement curve of unlabeled DHA.

#### Conformational BRET sensor

Raw BRET ratios were defined as acceptor emission/donor emission. The three BRET ratios prior epinephrine addition were averaged and defined as BRET_basal_. To quantify epinephrine-induced BRET changes, ΔBRET were calculated for each well and time point as a percent over basal ([(BRET_stim_− BRET_basal_)/BRET_basal_] × 100). Subsequently, the average ΔBRET of vehicle-treated control wells. The epinephrine concentration response curve was generated based on the vehicle-corrected ΔBRET measured 15 min after ligand addition.

Antagonist pK_B_ values were calculated using the agonist potency and concentration together with the antagonist IC_50_ values, using the Cheng-Prusoff equation. (Cheng and Prusoff, 1973; Leff and Dougall, 1993)

#### Nanobit complementation experiments

Four parameter concentration-response curves were fitted to individual experiments performed in duplicate, normalized to the 10 μM isoprenaline (100%) and vehicle control (0%) response at 31 min post agonist addition. From the isoprenaline potencies (EC_50_) in the absence and presence of Opto-prop-2, concentration ratios (CR) were calculated. The antagonist affinity (K_D_) was then estimated by a Schild plot of log [CR-1] against log [B] (the antagonist concentration) using the relationship:

$$\text{Log}\;\left[\text{CR}-1\right]=\text{Log}\;\left[\text{B}\right]-\text{Log}\;{\text{K}}_{\text{D}}.$$

#### Intact cardiomyocyte isolation and measurements

Changes in sarcomere length were recorded with a high-speed camera and Ionoptix software (Ionoptix, Westwood MA, USA). The contractility profiles were analyzed with the automated, batch analysis software ‘Transient Analysis Tools’ (CytoCypher, Amsterdam, NL). Fractional shortening of cardiomyocytes was compared between treatment conditions using one-way ANOVA with Dunnett’s multiple comparisons test.

## Data Availability

Data was analyzed using the proprietary software packages: Graphpad prism (GraphPad Software, San Diego CA, USA), Ionoptix software (Ionoptix, Westwood MA, USA) and ‘Transient Analysis Tools’ (CytoCypher, Amsterdam, NL).
