# Supplementary material for: Optical control of the β2-adrenergic receptor with opto-prop-2: A cis-active azobenzene analog of propranolol
Source: iScience. 2022 Aug 5;25(9):104882. doi: 10.1016/j.isci.2022.104882 (PMC9436767; doi:10.1016/j.isci.2022.104882)
Supplement: Document S1. Figures S1, S2, and Data S1 [file mmc1.pdf]

iScience, Volume *25*

Supplemental information

Optical control of the β_2-adrenergic receptor with opto-prop-2: A *cis*-active azobenzene analog of propranolol

Reggie Bosma, Nicola C. Dijon, Yang Zheng, Hannes Schihada, Niels J. Hauwert, Shuang Shi, Marta Arimont, Rick Riemens, Hans Custers, Andrea van de Stolpe, Henry F. Vischer, Maikel Wijtmans, Nicholas D. Holliday, Diederik W.D. Kuster, and Rob Leurs

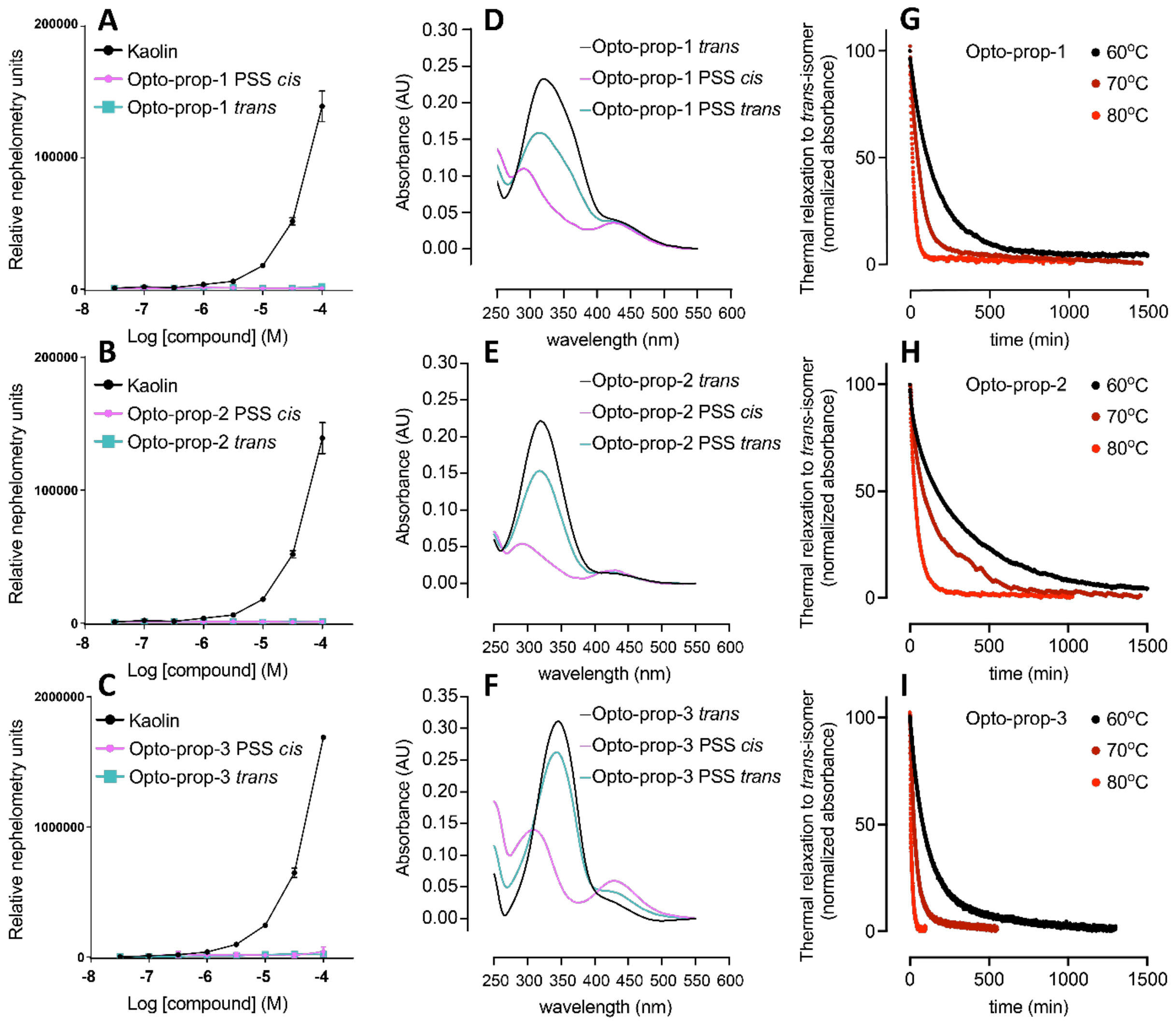

Figure S1. Photochemical characterization of Opto-prop-1, -2 and -3, related to Table 1 and Figure 2. (A - C) Nephelometry data of Optoprop-1, -2 and -3. Increasing concentrations of *trans*-Optoprop-1, -2 and -3 and PSS_{360} were measured together with Kaolin (control suspension) in aqueous buffer at physiological pH containing 1% DMSO. Data points represent the mean ± SD of triplicate values. (D - E) UV spectroscopy analysis of a sample of *trans*-Optoprop-1, -2 and -3 (25 µM in 1 % DMSO/HBBS buffer, black line) upon illumination with 360 nm (4-5 min) to PSS_{360} containing high levels of *cis* isomer (magenta line) and subsequent illumination with 434 nm (2-5 min) to PSS_{434}, containing mostly *trans* isomer (teal line). (G - I) Thermal relaxation of the *cis* isomer to the more stable *trans* isomer. The thermal relaxation of PSS_{360} is measured by monitoring the absorbance at 320 nm, 320 nm and 347 nm of Optoprop-1, -2 and -3, respectively.

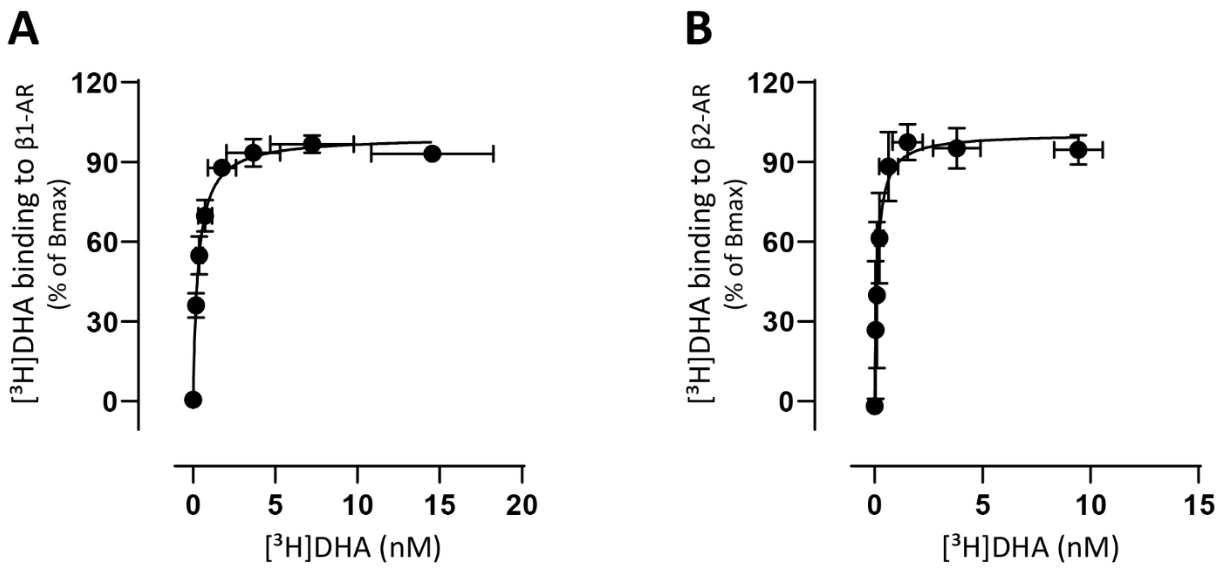

Figure S2. Saturation binding of [^{3}H]dihydroalprenolol to the β_1 and β_2 adrenergic receptors, related to Figure 3. Increasing concentrations [^{3}H]dihydroalprenolol were incubated with membranes of HEK293T cells transiently expressing either the β_1-AR (A) or β_2-AR (B). Both the radioligand concentration and bound radioligand were quantified by scintillation counting. Data represent the pooled mean $\pm$ SD of 4 experiments. Binding affinities (pK_d) of [^{3}H]dihydroalprenolol were determined by fitting the data in each individual experiment. [^{3}H]dihydroalprenolol has a pK_d value of 9.4 $\pm$ 0.1 and a B_{max} value of 56 $\pm$ 16 pmol/mg at membranes expressing the β_1-AR and a pK_d value of 10.0 $\pm$ 0.1 and a B_{max} value of 33 $\pm$ 19 pmol/mg at membranes expressing the β_2-AR.

Data S1. Synthesis procedures, related to Figure 1 and Scheme 1.

Unless mentioned otherwise, all reactions were performed under N_2 atmosphere. All chemicals and solvents were obtained from commercial suppliers (primarily Sigma-Aldrich, Acros Organics, Fluorochem and Combi-Blocks) and used without purification. DCM, DMF, THF and Et_2O were dried by passing through a PureSolv solvent purification system. Reactions were monitored by thin layer chromatography (Merck Silicagel 60 F254) by visualization under 254 nm lamp or under natural light conditions (for colored compounds). Flash column chromatography was performed with SNAP KP-Sil 50 µm (Biotage) or GraceResolv (Büchi) cartridges on Isolera One with UV-Vis detection (Biotage). Nuclear magnetic resonance (NMR) spectra were determined with a Brücker Avance 500 Ultrashield or a Brücker Avance 600 Ultrashield plus spectrometer. Chemical shifts are reported in parts per million (ppm) against the reference compound using the signal of the residual non-deuterated solvent ($CDCl_3$ δ = 7.26 ppm (^1H), δ = 77.16 ppm (^{13}C). NMR spectra were processed using MestreNova 14.1.1 software. The peak multiplicities are defined as follows: s, singlet; d, doublet; t, triplet; q, quartet; dd, doublet of doublets; ddd, doublet of doublets of doublets; dt, doublet of triplets; dq, doublet of quartets; td, triplet of doublets; tt, triplet of triplets; br, broad signal; m, multiplet; app, apparent. Melting trajectories were measured on a Buchi Melting Point M-565. Purity determination was performed with Liquid Chromatography using a Shimadzu LC-20AD liquid chromatography pump system with a Shimadzu SPDM20A photodiode array detector and MS detection with a Shimadzu LCMS-2010EV mass spectrometer operating in both positive and negative ionization mode. A Waters XBridge C18 column 5 µm 4.6x50 mm was used at 40ºC. The mobile phase used was a mixture of A = Water + 0.1% HCO_2H and B = acetonitrile (MeCN) + 0.1% HCO_2H. The eluent program used is as follows: flow rate: 1.0 mL/min, start 95% A in a linear gradient to 10% A over 4.5 min, hold 1.5 min at 10% A, in 0.5 min in a linear gradient to 95% A, hold 1.5 min at 95% A, total runtime: 8.0 min. Compound purities were calculated as the percentage peak area of the analyzed compound by UV detection at 254 nm. All chemistry and analyses of photosensitive compounds were carried out under dimmed or red light. High-resolution mass spectra (HRMS) were recorded on a Bruker micrOTOF mass spectrometer using ESI in positive ion mode (HRMS).

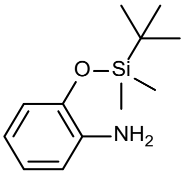

2-((*Tert*-butyldimethylsilyl)oxy)aniline (2a), related to Figure 1 and Scheme 1.
To a stirred solution of 2-aminophenol **1a** (3.00 g, 27.5 mmol) and imidazole (2.81 g, 41.2 mmol) in DMF (30 mL) at 0°C, TBMDS-Cl (4.97 g, 33.0 mmol) dissolved in DMF (16 mL) was added in a dropwise fashion. After stirring at RT for 3 h, the reaction mixture was poured into ice-cold brine (250 mL). The mixture was extracted using EtOAc (3x 50 mL). The combined organic phases were washed with ice-cold brine (100 mL), dried over Na_2SO_4, filtered and evaporated *in vacuo*. The product was purified using FC eluting with a gradient from cyclohexane to cyclohexane:EtOAc 15:1 to yield the title compound as a colorless oil (6.12 g, 99%). ^1H NMR (500 MHz, $CDCl_3$) δ 6.82 – 6.77 (m, 1H), 6.77 – 6.73 (m, 2H), 6.65 (ddd, *J* = 7.9, 7.2, 1.8 Hz, 1H), 3.87 – 3.43 (br, 2H), 1.03 (s, 9H), 0.25 (s, 6H); ^{13}C NMR (126 MHz, $CDCl_3$) δ 143.2, 138.0, 121.9, 118.8, 118.6, 116.0, 26.0, 18.4, -4.1; LC-MS: t_r= 5.30 min, purity: 76.5%, M/z $[M+H]^+$ 224.

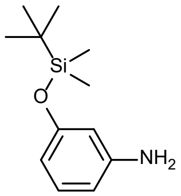

3-((*Tert*-butyldimethylsilyl)oxy)aniline (2b), related to Figure 1 and Scheme 1.
Imidazole (3.99 g, 58.6 mmol), 3-aminophenol **1b** (4.00 g, 36.7 mmol) and TBDMS-Cl (7.18 g, 47.7 mmol) were mixed with THF (100 mL). The mixture was stirred vigorously overnight at RT. The solution was concentrated *in vacuo*. To the residue was added H_2O (100 mL). The mixture was extracted using Et_2O (3x100 mL). The combined organic phases were dried over Na_2SO_4, filtered and evaporated *in vacuo*. The crude product was purified using FC eluting isocratically with cyclohexane:EtOAc 9:1 to yield the title compound as a colorless oil (8.12 g, 99%). ^{1}H NMR (600 MHz, $CDCl_3$) δ 6.99 (app t, *J* = 8.0 Hz, 1H), 6.31 (ddd, *J* = 8.0, 2.2, 0.9 Hz, 1H), 6.26 (ddd, *J* = 8.1, 2.2, 0.9 Hz, 1H), 6.21 (app t, *J* = 2.2 Hz, 1H), 3.78 (s, 2H), 0.97 (s, 9H), 0.19 (s, 6H); ^{13}C NMR (151 MHz, $CDCl_3$) δ 156.8, 147.5, 130.1, 110.8, 108.8, 107.4, 25.8, 18.3, -4.2; LC-MS: t_r= 4.94 min, purity: >99%, M/z $[M+H]^+$ 224.

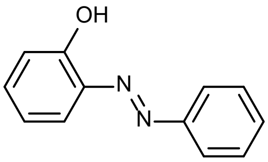

(*E*)-2-(phenyldiazenyl)phenol (3a), related to Figure 1 and Scheme 1.
To a stirred solution of PhNO (1.44 g, 13.0 mmol) in AcOH (67 mL) was added aniline 2a (3.00 g, 13.0 mmol). The mixture was stirred at RT overnight. The solution was evaporated *in vacuo* and co-evaporated twice using EtOAc. THF (50 mL) was added and the solution was cooled to 0 °C. TBAF in THF (1.0 M, 13.4 mL, 13.4 mmol) was added dropwise and the solution was stirred for 30 min. Aq. sat. $NaHCO_3$ (30 mL) was added. After 30 min of stirring, EtOAc (30 mL) was added. The layers were partitioned. The organic phase was washed with aq. sat. $NaHCO_3$ (20 mL) and brine (20 mL), dried over Na_2SO_4, filtered and evaporated *in vacuo*. The crude product was purified using FC eluting with a gradient from cyclohexane to cyclohexane: EtOAc 4:1 to yield the title compound as a red solid (1.35 g, 51 %). ^{1}H NMR (500 MHz, $CDCl_3$) δ 12.95 (s, 1H), 7.96 (dd, *J* = 7.9, 1.7 Hz, 1H), 7.91 – 7.86 (m, 2H), 7.56 – 7.45 (m, 3H), 7.36 (ddd, *J* = 8.2, 7.2, 1.7 Hz, 1H), 7.08 (ddd, *J* = 8.2, 7.3, 1.3 Hz, 1H), 7.04 (dd, *J* = 8.2, 1.3 Hz, 1H); ^{13}C NMR (126 MHz, $CDCl_3$) δ 152.9, 150.6, 137.5, 133.4, 133.4, 131.3, 129.5, 122.4, 120.1, 118.3; LC-MS: t_r= 5.37 min, purity: >99%, M/z $[M+H]^+$ 199.

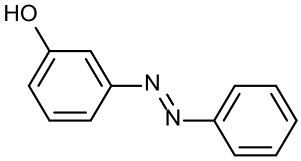

(*E*)-3-(phenyldiazenyl)phenol (3b), related to Figure 1 and Scheme 1.
To a stirred solution of PhNO (0.240 g, 2.24 mmol) in AcOH (11 mL) was added aniline **2b** (0.500 g, 2.24 mmol). The mixture was stirred at RT overnight. The solution was evaporated *in vacuo* and co-evaporated twice using EtOAc. THF (10 mL) was added and the solution was cooled to 0 °C. TBAF in THF (1.0 M, 13.4 mL, 13.4 mmol) was added dropwise and the solution was stirred for 30 min. Aq. sat. $NaHCO_3$ (5 mL) was added. After 30 min of stirring, EtOAc (5 mL) was added. The layers were partitioned. The organic phase was washed with aq. sat. $NaHCO_3$ (5 mL) and brine (5 mL), dried over Na_2SO_4, filtered and evaporated *in vacuo*. The crude product was purified using FC eluting with a gradient from cyclohexane to cyclohexane: EtOAc 4:1 to yield the title compound as a red solid (0.312 g, 70%). ^{1}H NMR (500 MHz, $CDCl_3$) δ 7.94 – 7.89 (m, 2H), 7.59 – 7.46 (m, 4H), 7.41 (app t, *J* = 8.0 Hz, 1H), 7.38 (dd, *J* = 2.6, 1.7 Hz, 1H), 6.98 (ddd, *J* = 8.1, 2.6, 1.0 Hz, 1H), 5.02 (bs, 1H); ^{13}C NMR (126 MHz,

$CDCl_3$) δ 156.3, 154.0, 152.6, 131.3, 130.3, 129.3, 123.0, 118.3, 117.7, 107.8; LC-MS: t_r= 4.53 min, purity: >99%, M/z $[M+H]^+$ 199.

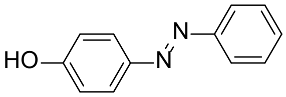

(*E*)-4-(phenyldiazenyl)phenol (3c), related to Figure 1 and Scheme 1.
4-Aminophenol **1c** (3.00 g, 27.5 mmol) was added to a solution of PhNO (2.95 g, 27.5 mmol) in AcOH (100 mL). The reaction mixture was stirred at RT for 24 h. The reaction mixture was evaporated *in vacuo*. The crude material was purified using FC eluting isocratically with cyclohexane:EtOAc 9:1 to yield the title product as an orange solid (1.42 g, 26%). ^{1}H NMR (600 MHz, $CDCl_3$) δ 7.92 - 7.86 (m, 4H), 7.54 - 7.48 (m, 2H), 7.47 - 7.42 (m, 1H), 6.98 - 6.93 (m, 2H), 5.29 (br s, 1H). ^{13}C NMR (151 MHz, $CDCl_3$) δ 158.5, 152.7, 147.3, 130.6, 129.2, 125.2, 122.7, 116.0. LC-MS: t_r= 4.54 min, purity: >99%, M/z $[M+H]^+$ 199.

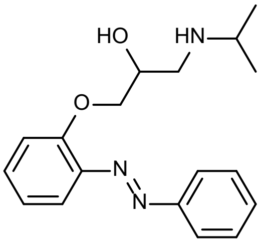

***Rac*-(*E*)-1-(isopropylamino)-3-(2-(phenyldiazenyl)phenoxy)propan-2-ol (4a, Opto-prop-1, VUF17061), related to Figure 1 and Scheme 1.**
Phenol **3a** (0.200 g, 1.01 mmol) was dissolved in EtOH (0.6 mL). Powdered NaOH (0.048 g, 1.21 mmol) was added. After 10 min of stirring at RT, *rac*-epichlorohydrin (0.237 mL, 3.03 mmol) was added. The reaction mixture was stirred at 50 °C overnight in a closed microwave vial. After cooling to RT, *i*PrNH_2 (0.867 mL, 10.1 mmol) was added and the mixture was stirred at RT for 1 h. The reaction mixture was concentrated *in vacuo* and the mixture was partitioned between aq. sat Na_2CO_3 (5 mL) and EtOAc (5 mL). The organic phase was washed with brine (5 mL), dried over Na_2SO_4, filtered and evaporated *in vacuo*. The compound was purified using FC with a gradient using cyclohexane:EtOAc:TEA 50:45:5 to EtOAc:MeOH:TEA 90:5:5 to yield the title compound as an orange solid (104 mg, 33%). Melting trajectory: 86.2-98.7 °C. ^{1}H NMR (600 MHz, DMSO-d_6) δ 7.89 – 7.84 (m, 2H), 7.60 – 7.53 (m, 4H), 7.51 (ddd, *J* = 8.8, 7.2, 1.7 Hz, 1H), 7.30 (dd, *J* = 8.4, 1.2 Hz, 1H), 7.05 (ddd, *J* = 8.1, 7.3, 1.2 Hz, 1H), 5.01 (br s, 1H), 4.19 – 4.09 (m, 2H), 3.94 (app p, *J* = 5.5 Hz, 1H), 3.32 – 3.26 (m, 1H), 2.80 (dd, *J* = 11.7, 4.7 Hz, 1H), 2.71 (hept, *J* = 6.2 Hz, 1H), 2.63 (dd, *J* = 11.8, 7.0 Hz, 1H), 0.96 (d, *J* = 6.2 Hz, 3H), 0.95 (d, *J* = 6.2 Hz, 3H); ^{13}C NMR (151 MHz, DMSO-d_6) δ 156.4, 152.4, 141.7, 133.0, 131.2, 129.4, 122.5, 120.8, 116.3, 115.2, 72.1, 68.3, 50.0, 48.2, 22.8; LC-MS: t_r= 3.39 min, purity: 99%, M/z $[M+H]^+$ 314; HRMS: calc. for $C_{18}H_{24}N_3O_2^+$ $[M+H]^+$: 314.1863, found: 314.1859.

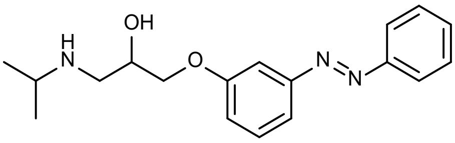

***Rac*-(*E*)-1-(isopropylamino)-3-(3-(phenyldiazenyl)phenoxy)propan-2-ol (4b, Opto-prop-2, VUF17062), related to Figure 1 and Scheme 1.**
Phenol **3b** (0.200 g, 1.01 mmol) was dissolved in EtOH (0.6 mL). Powdered NaOH (0.048 g, 1.21 mmol) was added. After 10 min of stirring at RT, *rac*-epichlorohydrin (0.237 mL, 3.03 mmol) was added. The reaction mixture was stirred at 50 °C overnight in a closed microwave vial. After cooling to RT, *i*PrNH_2 (0.867 mL, 10.1 mmol) was added and the mixture was stirred at RT for 1 h. The reaction mixture was concentrated *in vacuo* and the mixture was partitioned between aq. sat Na_2CO_3 (5 mL) and EtOAc (5

mL). The organic phase was washed with brine (5 mL), dried over Na_2SO_4, filtered and evaporated *in vacuo*. The compound was purified using FC with a gradient using cyclohexane:EtOAc:TEA 50:45:5 to EtOAc:MeOH:TEA 90:5:5 to yield the title compound as an orange solid (69 mg, 22%). Melting trajectory: 96.3-104.2 °C. ^{1}H NMR (600 MHz, CD_3OD) δ 7.93 – 7.88 (m, 2H), 7.61 – 7.46 (m, 6H), 7.16 (ddd, *J* = 8.2, 2.6, 1.0 Hz, 1H), 4.33 – 4.26 (m, 1H), 4.19 – 4.10 (m, 2H), 3.48 (app p, *J* = 6.6 Hz, 1H), 3.32 (dd, *J* = 12.7, 9.6 Hz, 1H), 3.19 (dd, *J* = 12.7, 9.6 Hz, 1H), 1.39 (d, *J* = 6.4 Hz, 3H), 1.38 (d, *J* = 6.4 Hz, 3H); ^{13}C NMR (151 MHz, CD_3OD) δ 160.7, 155.2, 153.9, 132.5, 131.2, 130.3, 123.8, 119.1, 118.7, 107.6, 71.2, 66.9, 52.1, 48.4, 19.4, 18.8; LC-MS: t_r= 3.53 min, purity: 97%, M/z $[M+H]^+$ 314; HRMS: calc. for $C_{18}H_{24}N_3O_2^+$ $[M+H]^+$: 314.1863, found: 314.1858.

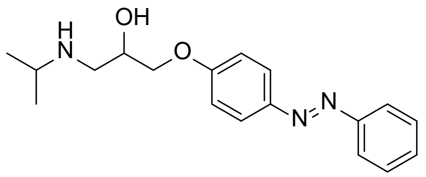

***Rac*-(*E*)-1-(isopropylamino)-3-(4-(phenyldiazenyl)phenoxy)propan-2-ol (4c, Opto-prop-3, VUF25417), related to Figure 1 and Scheme 1.**

Phenol **3c** (0.250 g, 1.26 mmol) was dissolved in EtOH (0.6 mL). Powdered NaOH (0.053 g, 1.05 mmol) was added. After 10 min of stirring at RT, *rac*-epichlorohydrin (0.296 mL, 3.78 mmol) was added. The reaction mixture was stirred at 50 °C overnight in a closed microwave vial. After cooling to RT, *i*$PrNH_2$ (1.10 mL, 12.6 mmol) was added and the mixture was stirred at RT for 1 h. The reaction mixture was concentrated *in vacuo* and the mixture was partitioned between aq. sat Na_2CO_3 (5 mL) and EtOAc (5 mL). The organic phase was washed with brine (5 mL), dried over Na_2SO_4, filtered and evaporated *in vacuo*. The compound was purified using FC with a gradient using cyclohexane:EtOAc:TEA 50:45:5 to EtOAc:MeOH:TEA 90:5:5 to yield the title compound as an orange solid (162 mg, 41%). Melting trajectory: 108.6-109.9 °C ^{1}H NMR (600 MHz, DMSO-d_6) δ 7.90 - 7.87 (m, 2H), 7.86 - 7.82 (m, 2H), 7.59 - 7.55 (m, 2H), 7.54 - 7.50 (m, 1H), 7.16 - 7.12 (m, 2H), 5.03 (br d, *J* = 4.0 Hz, 1H), 4.09 (dd, *J* = 9.8, 4.3 Hz, 1H), 3.99 (dd, *J* = 9.8, 6.2 Hz, 1H), 3.91 - 3.84 (m, 1H), 2.73 - 2.66 (m, 2H), 2.58 (dd, *J* = 11.6, 6.7 Hz, 1H), 1.53 (br s, 1H), 0.99 (d, *J* = 2.8 Hz, 3H), 0.98 (d, *J* = 2.8 Hz, 3H). ^{13}C NMR (151 MHz, DMSO-d_6) δ 161.6, 152.0, 146.1, 130.8, 129.4, 124.6, 122.2, 115.1, 71.3, 68.4, 49.9, 48.2, 23.0, 23.0. LC-MS: t_r= 3.68 min, purity: >99%, M/z $[M+H]^+$ 314; HRMS: calc. for $C_{18}H_{24}N_3O_2^+$ $[M+H]^+$: 314.1863, found: 314.1864.

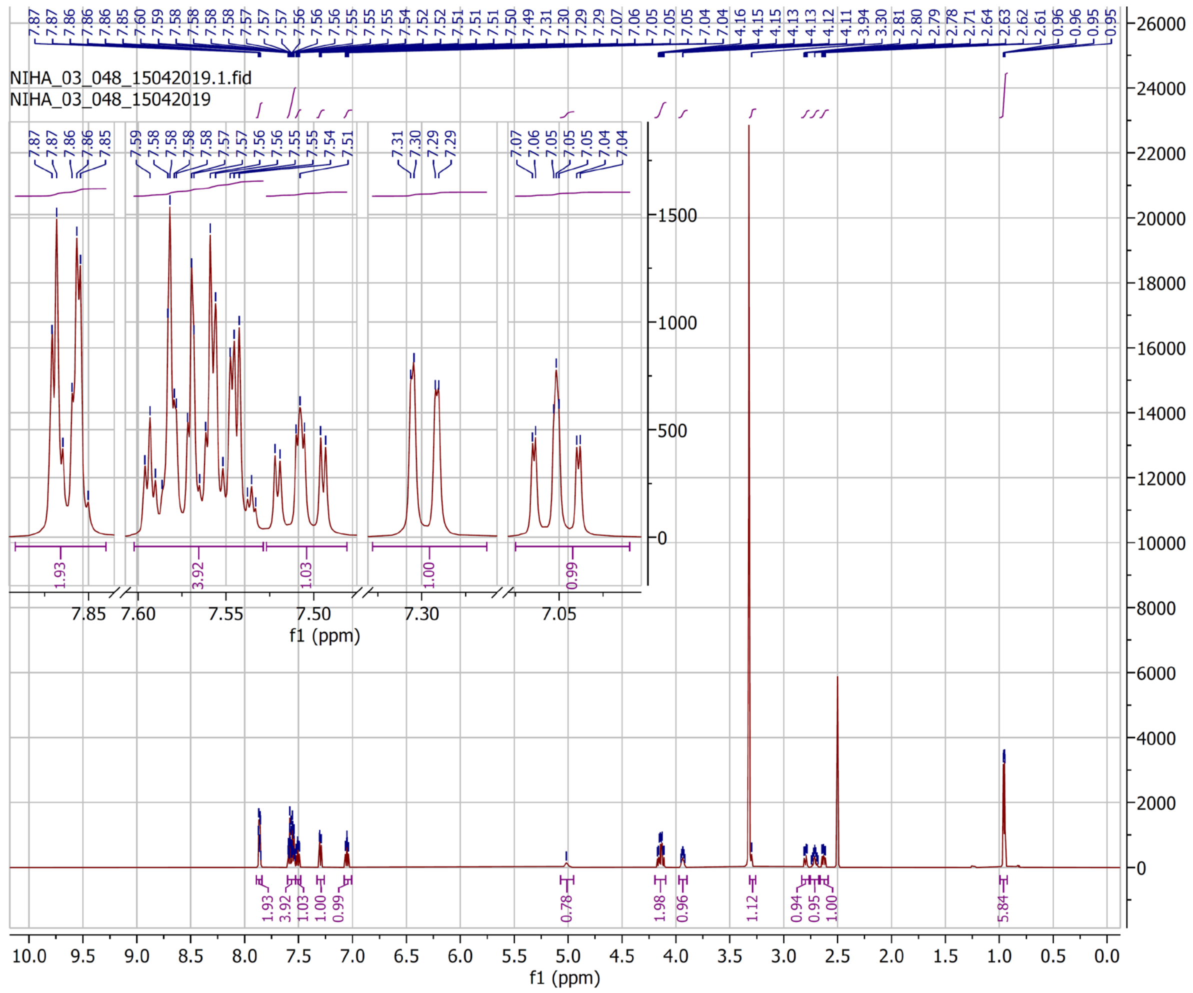

^{1}H NMR spectrum of *trans*- Optoprop-1 in DMSO-d_6, related to Figure 1 and Scheme 1.

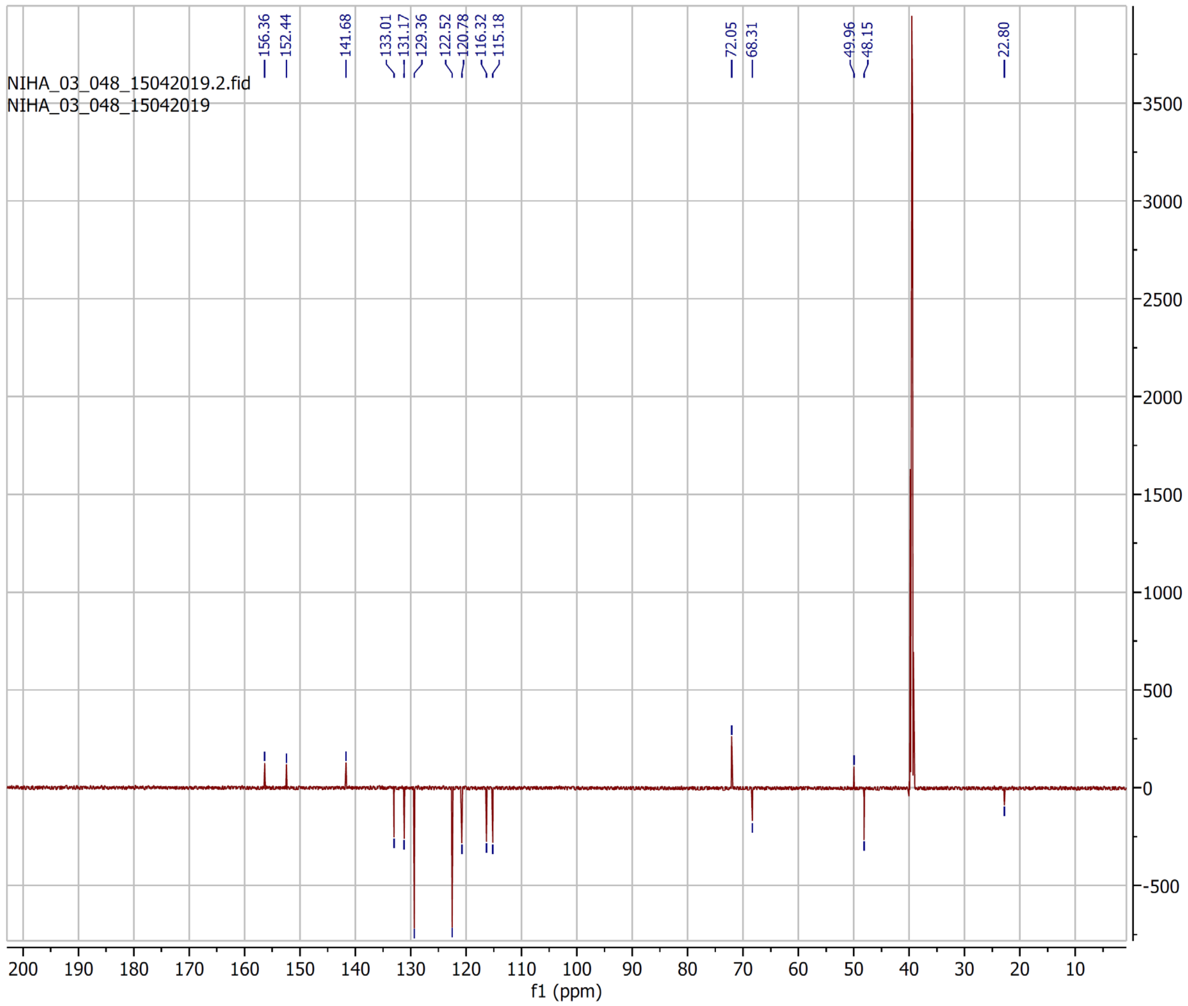

^{13}C NMR spectrum of *trans*- Optoprop-1 in DMSO-d_6, related to Figure 1 and Scheme 1.

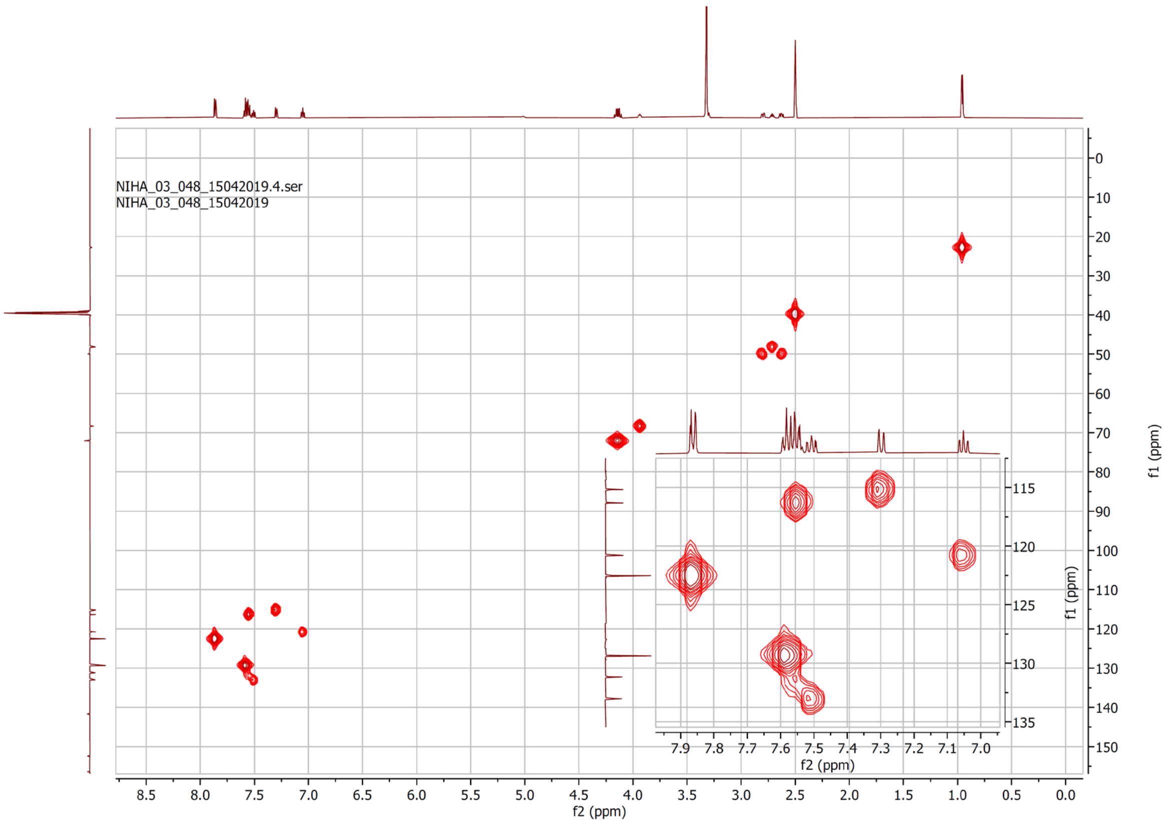

HSQC spectrum of *trans*-Optoprop-1 in DMSO-d$_6$, related to Figure 1 and Scheme 1.

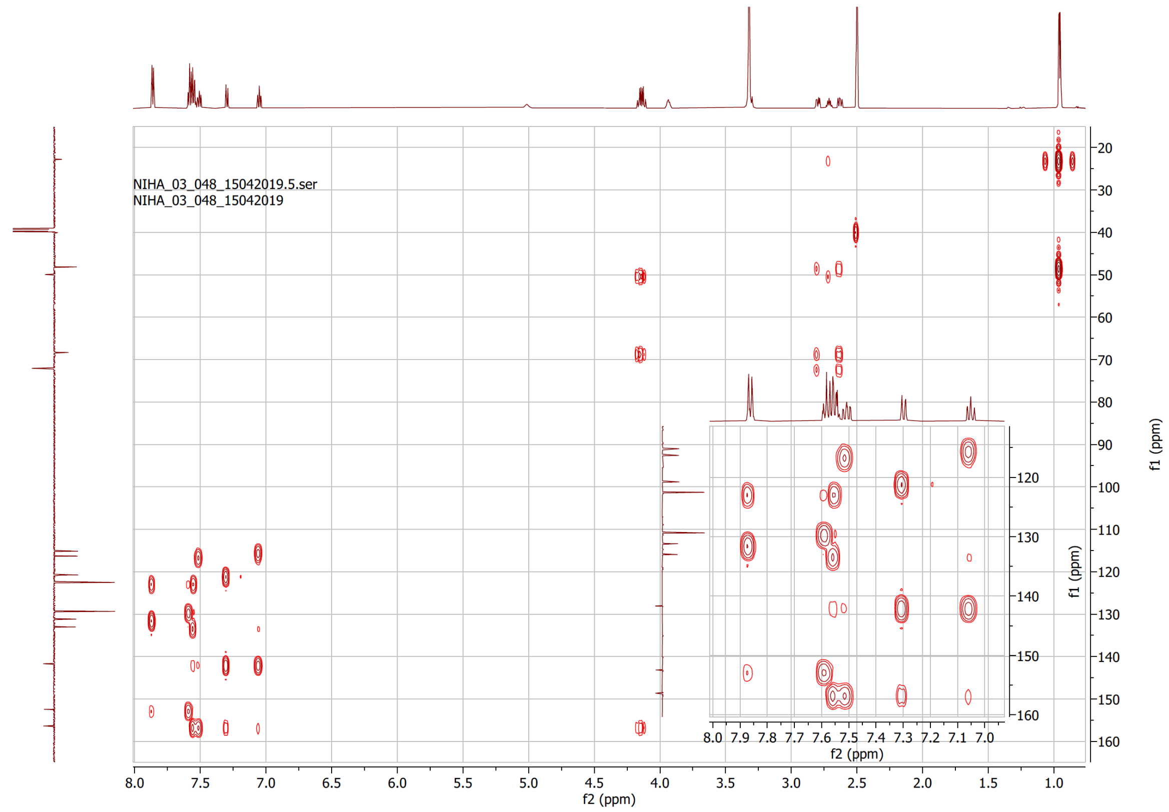

HMBC spectrum of *trans*- Optoprop-1 in DMSO-d_6, related to Figure 1 and Scheme 1.

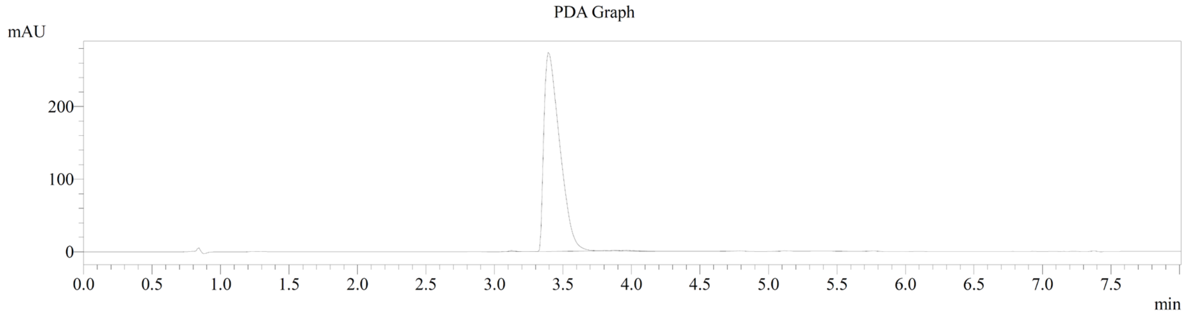

PeakTable

PDA Ch1 254nm 4nm

Peak#	Name	Ret. Time	Area	Area %
1		3.120	4340	0.195
2		3.390	2210706	99.337
3		3.955	10411	0.468

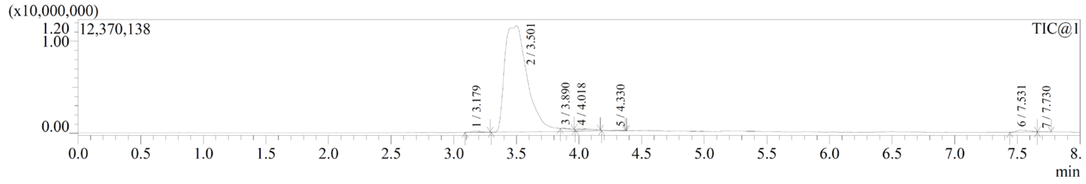

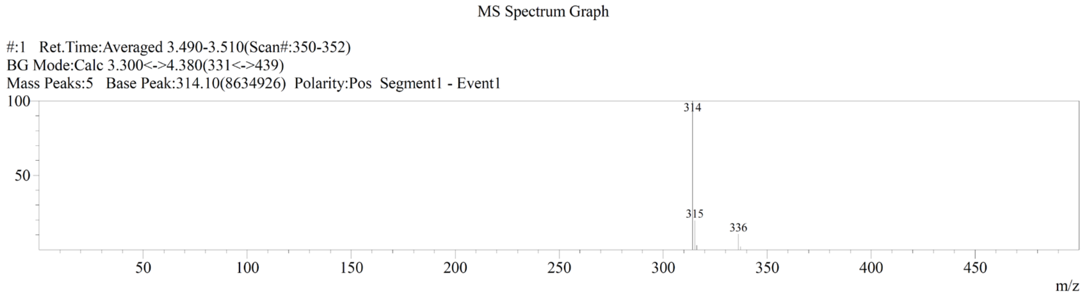

LCMS analysis of *trans*- Optoprop-1, related to Figure 1 and Scheme 1.

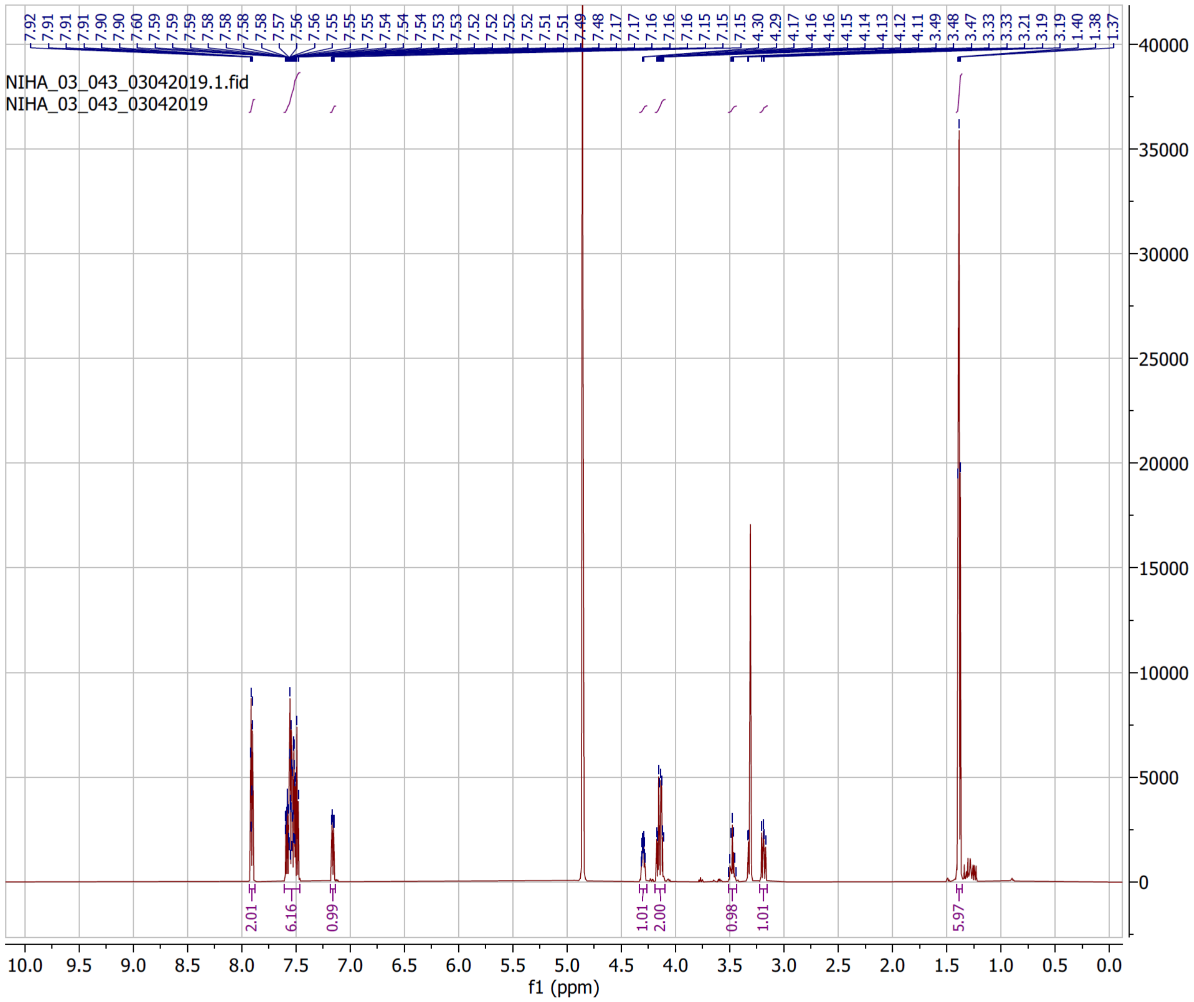

^{1}H NMR spectrum of *trans*-Optoprop-2 in CD_3OD, related to Figure 1 and Scheme 1.

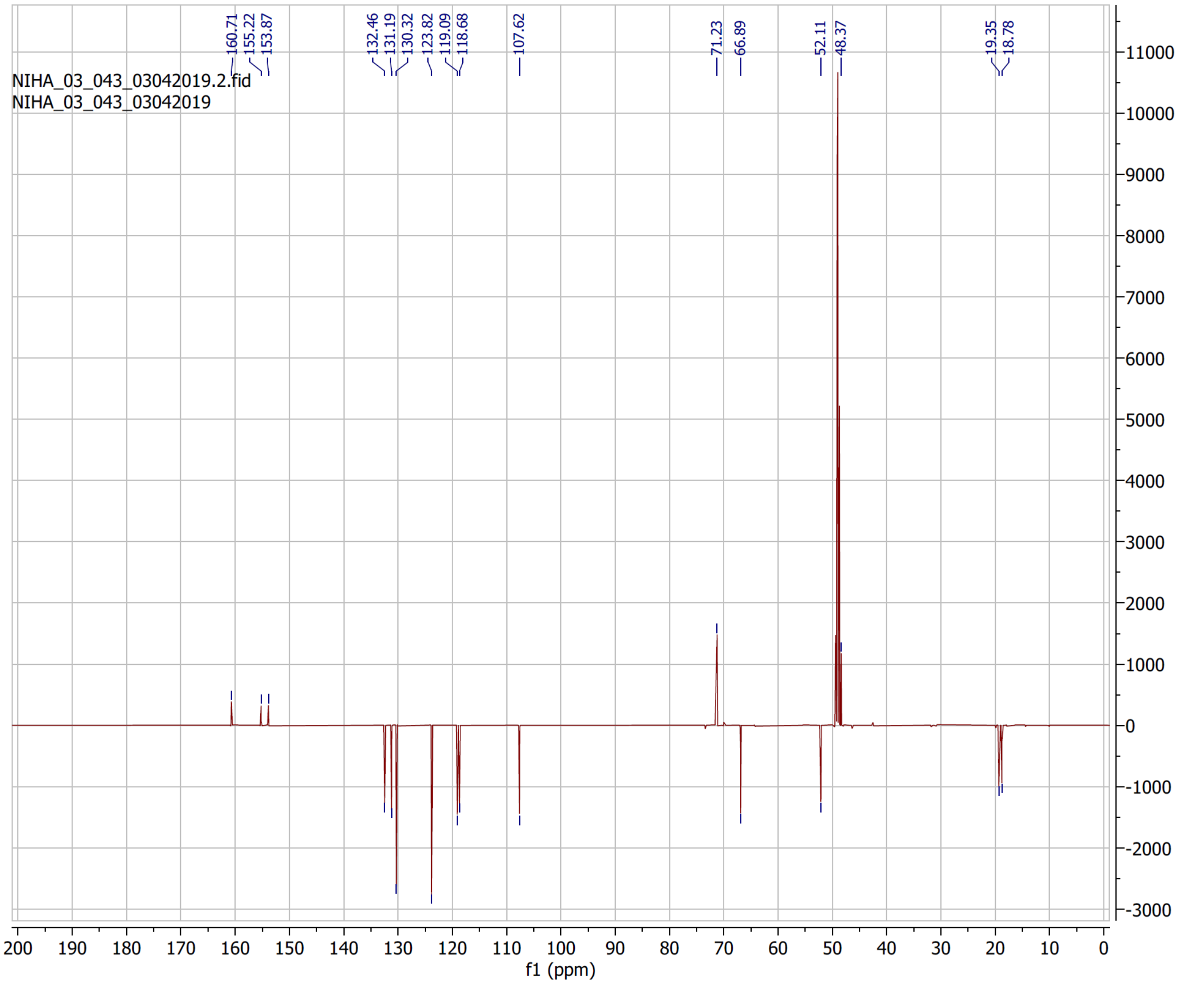

^{13}C NMR spectrum of *trans*-Optoprop-2 in CD_3OD, related to Figure 1 and Scheme 1.

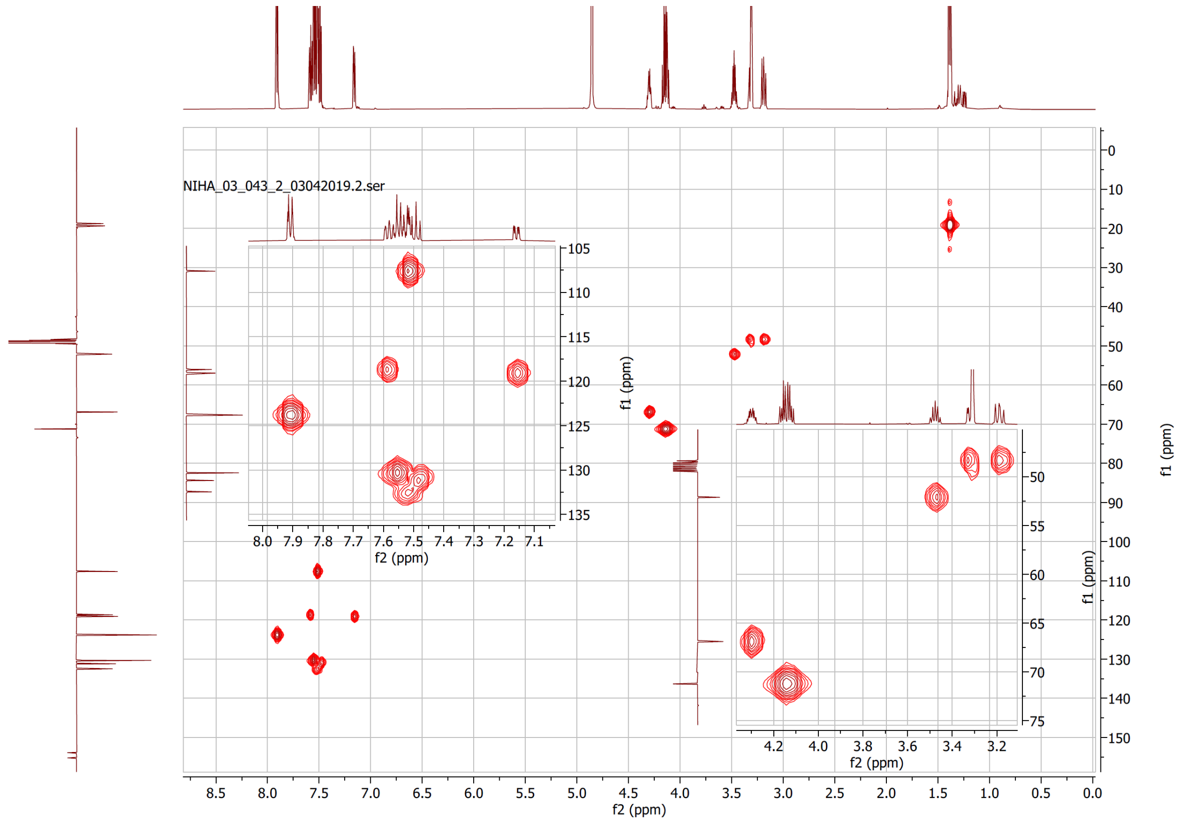

HSQC spectrum of *trans*-Optoprop-2 in CD_3OD, related to Figure 1 and Scheme 1.

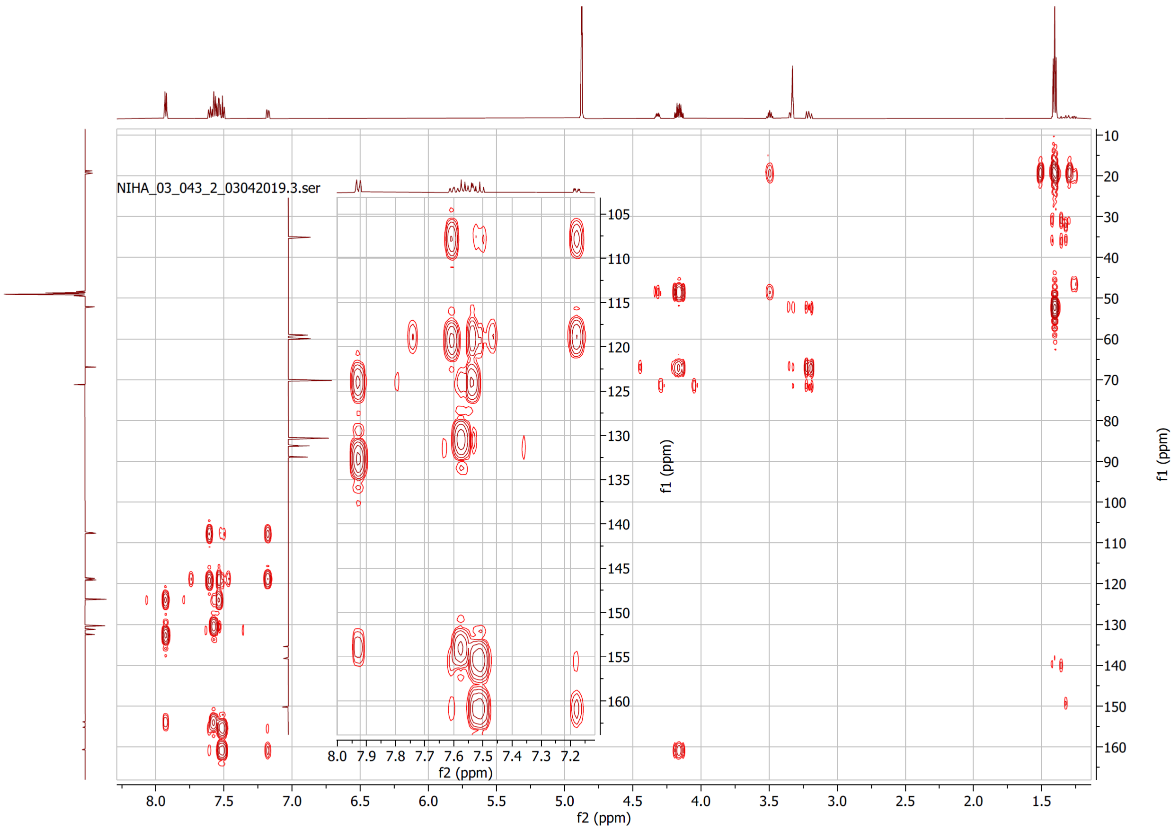

HMBC spectrum of *trans*-Optoprop-2 in CD_3OD, related to Figure 1 and Scheme 1.

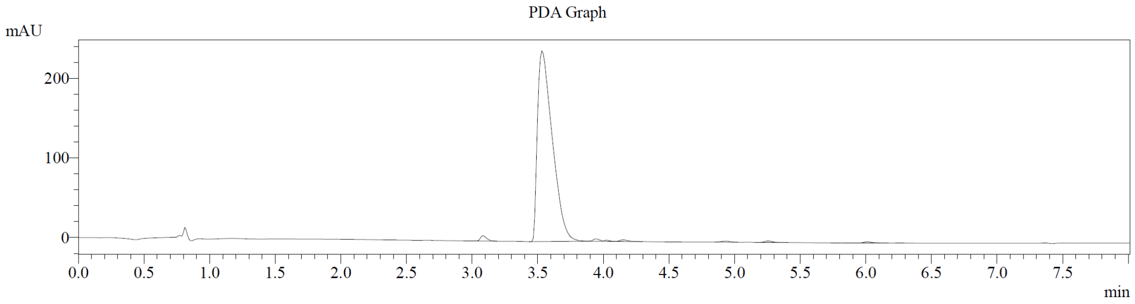

PeakTable

PDA Ch1 254nm 4nm

Peak#	Name	Ret. Time	Area	Area %
1		3.078	22142	1.131
2		3.528	1893547	96.681
3		3.939	22569	1.152
4		4.926	6216	0.317
5		5.250	7962	0.407
6		6.005	6118	0.312

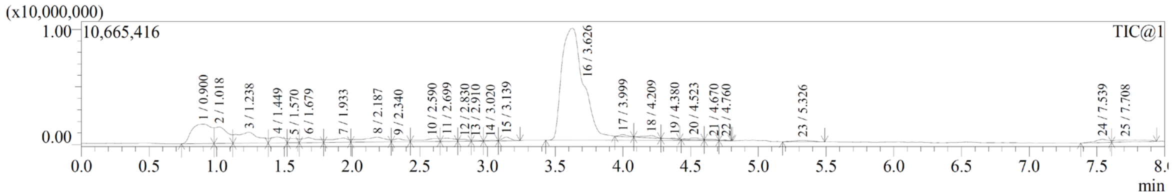

MS Spectrum Graph

#:1 Ret.Time:Averaged 3.620-3.640(Scan#:363-365)
BG Mode:Calc 3.430<->4.810(344<->482)
Mass Peaks:4 Base Peak:314.10(7684921) Polarity:Pos Segment1 - Event1

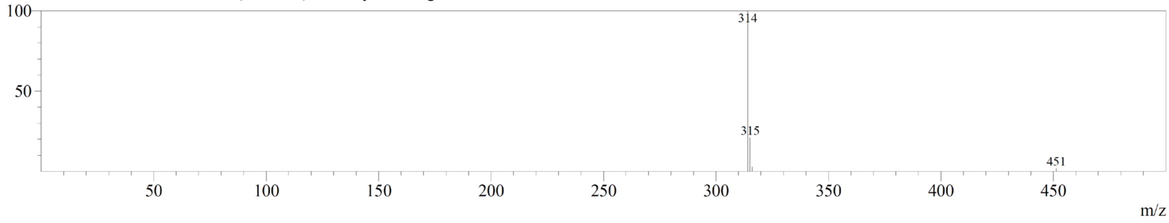

LCMS analysis of *trans*-Optoprop-2, related to Figure 1 and Scheme 1.

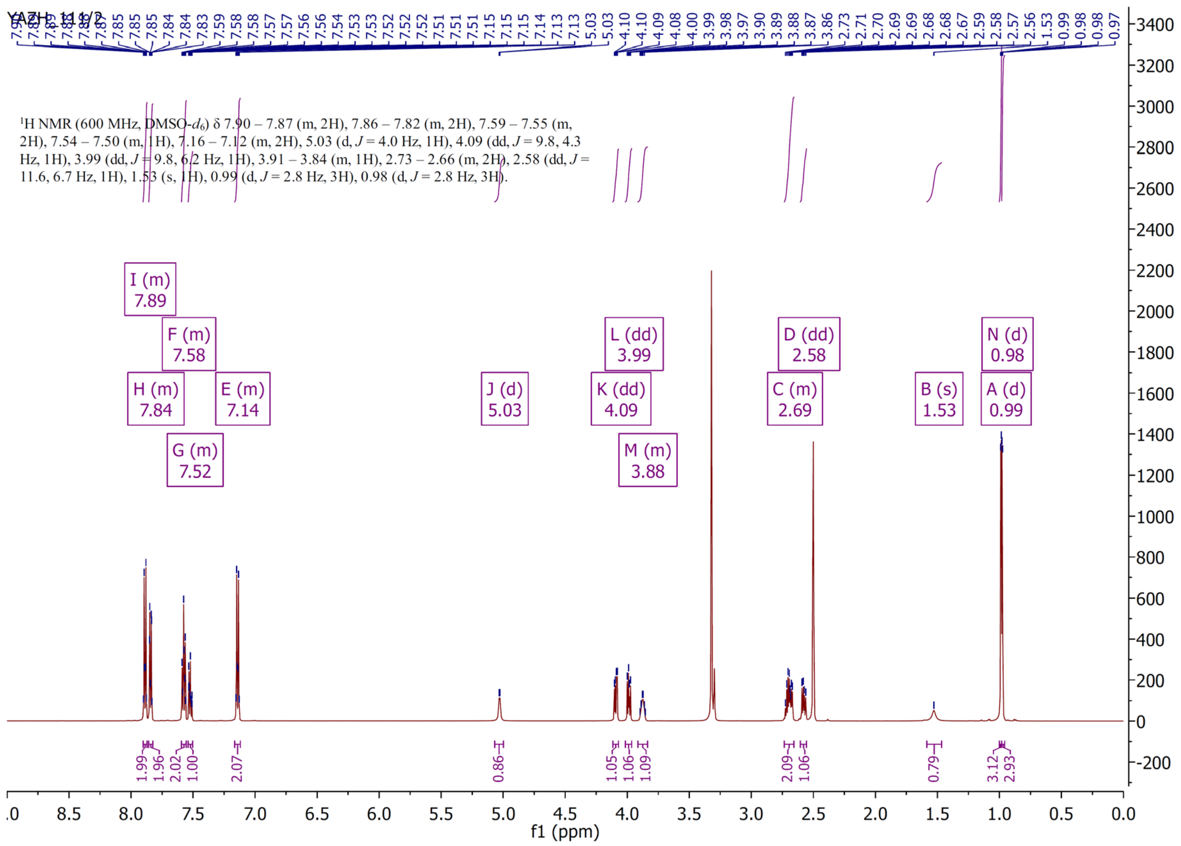

^{1}H NMR spectrum of *trans*-Optoprop-3 in DMSO-d$_6$, related to Figure 1 and Scheme 1.

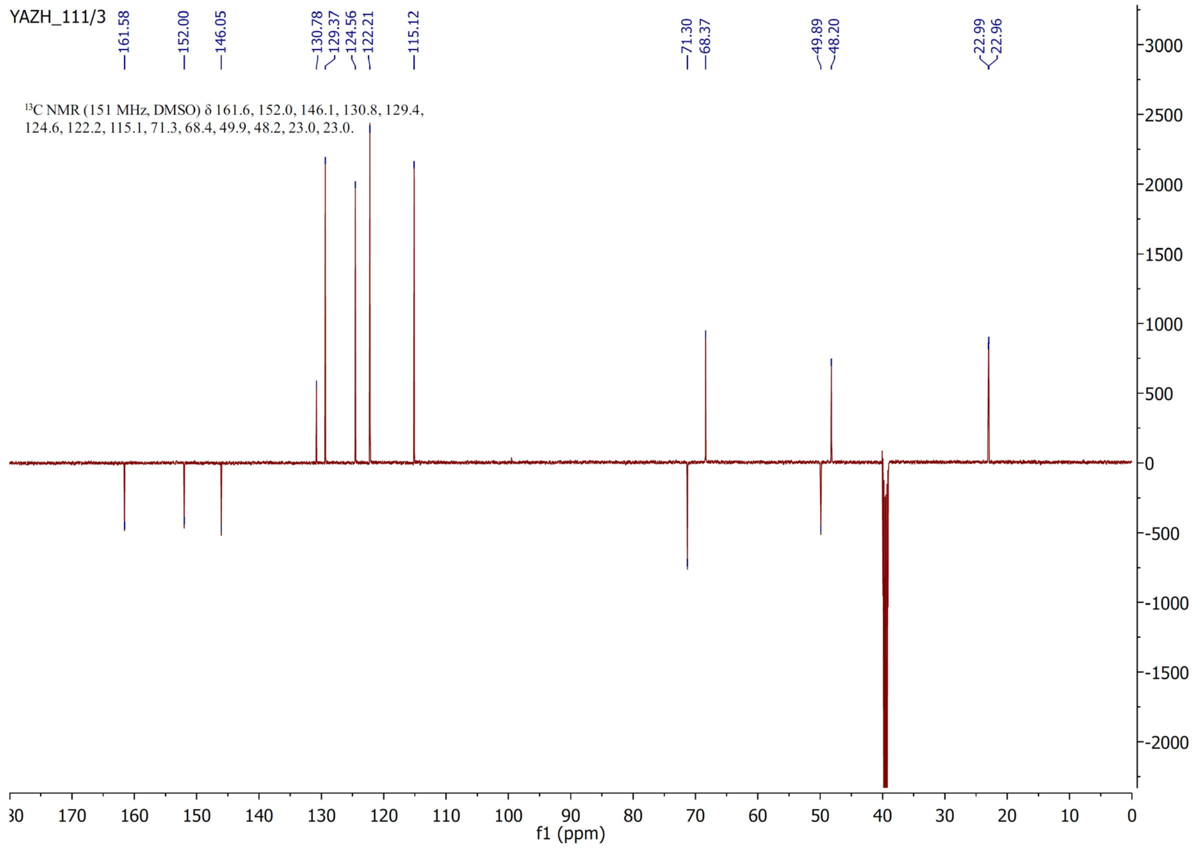

^{13}C NMR spectrum of *trans*-Optoprop-3 in DMSO-d_6, related to Figure 1 and Scheme 1.

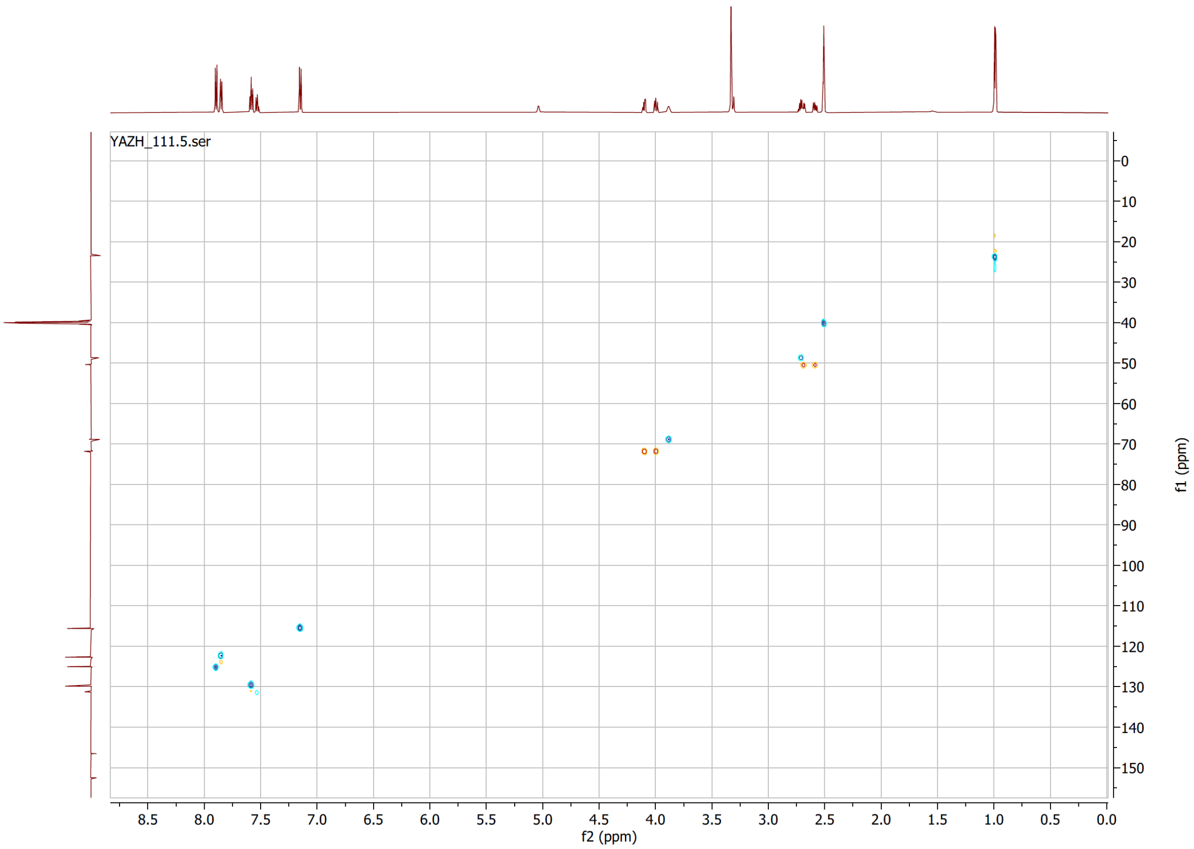

HSQC spectrum of *trans*-Optoprop-3 in DMSO-d_6, related to Figure 1 and Scheme 1.

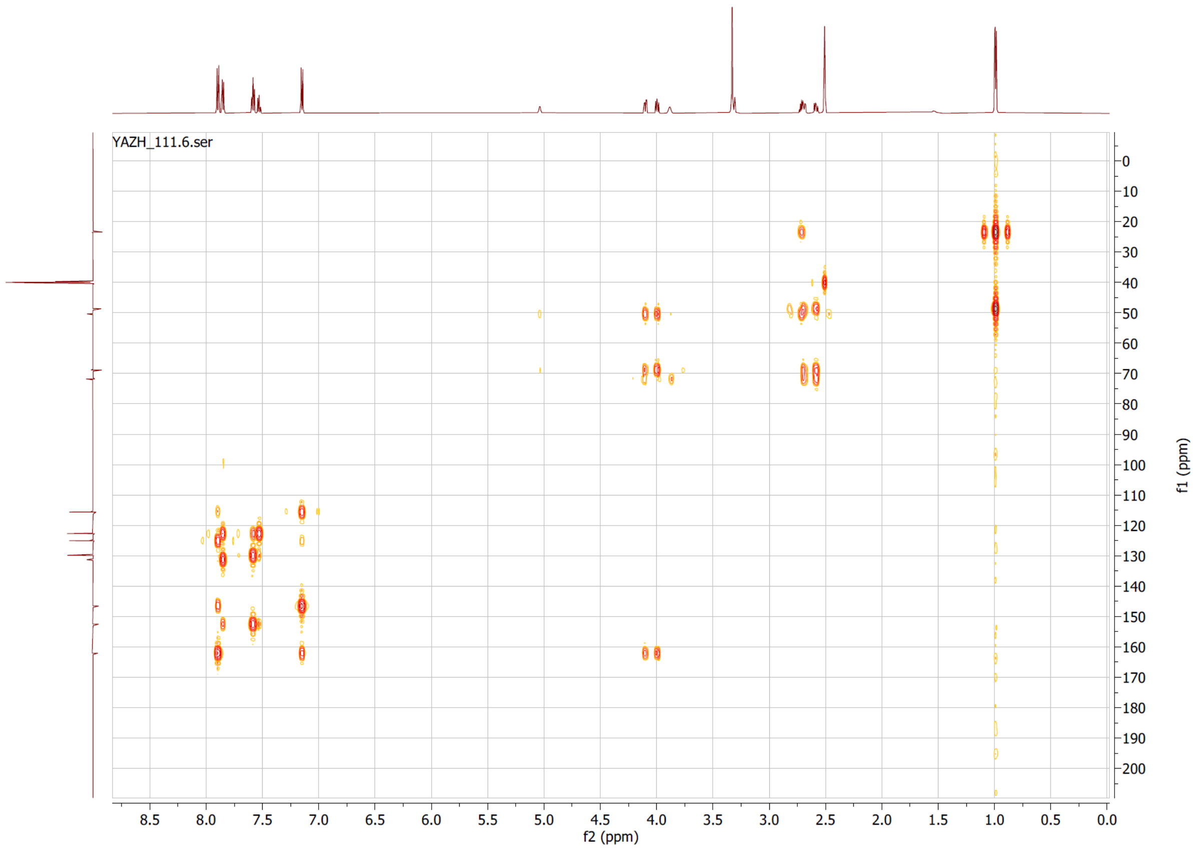

HMBC spectrum of *trans*-Optoprop-3 in DMSO-d_6, related to Figure 1 and Scheme 1.

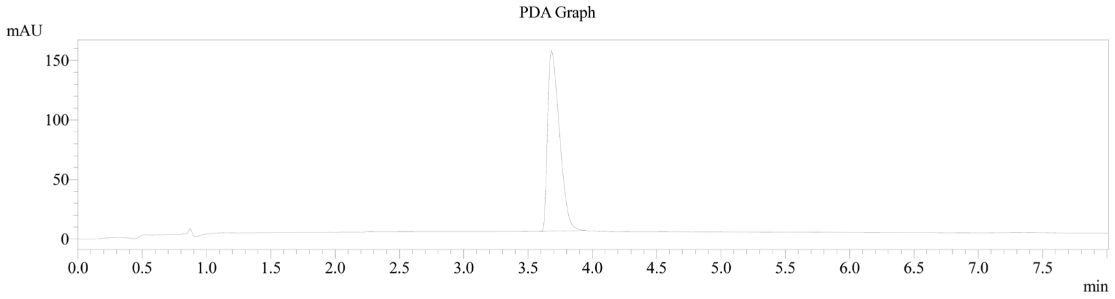

PeakTable

PDA Ch1 254nm 4nm

Peak#	Name	Ret. Time	Area	Area %
1		3.677	974940	100.000

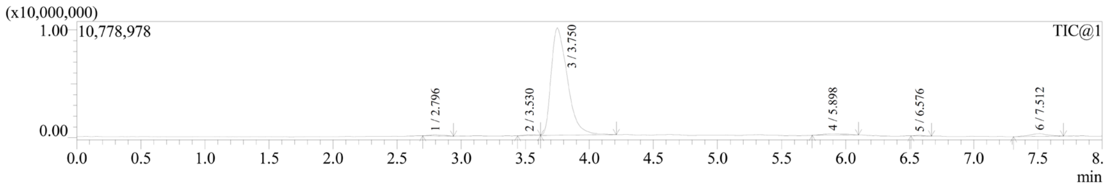

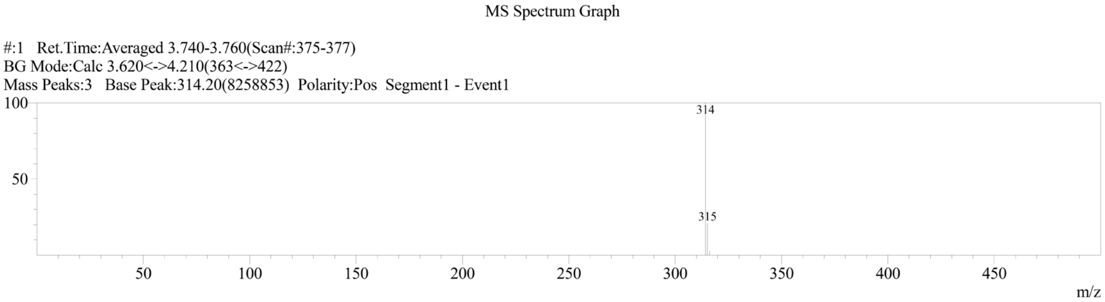

LCMS analysis of *trans*-Optoprop-3, related to Figure 1 and Scheme 1.
